# Steering Pt Cluster Dimensionality via the Surface Oxidation State of CeO_2_(111) Thin Films

**DOI:** 10.1021/acscatal.5c05570

**Published:** 2025-10-22

**Authors:** Johanna Reich, Mina Soltanmohammadi, Vedran Vonk, Sebastian Kaiser, Ueli Heiz, Andreas Stierle, Friedrich Esch, Barbara A. J. Lechner

**Affiliations:** † Functional Nanomaterials Group and Catalysis Research Center, Department of Chemistry, TUM School of Natural Sciences, 9184 Technical University of Munich, Garching 85748, Germany; ‡ Germany and Chair of Physical Chemistry and Catalysis Research Center, Department of Chemistry, TUM School of Natural Sciences, Technical University of Munich, Garching 85748, Germany; § Centre for X-Ray and Nano Science, 28332 Deutsches Elektronen-Synchrotron (DESY), Hamburg D-22607, Germany; ∥ Institute for Advanced Study, Technical University of Munich, Garching 85748, Germany; ⊥ Physics Department, University of Hamburg, Hamburg D-20355, Germany

**Keywords:** heterogeneous catalysis, ceria thin films, size-selected Pt clusters, O vacancies, sintering, 2D/3D growth, scanning tunneling microscopy

## Abstract

Ceria has recently regained attention in catalysis research, thanks to its ability to reversibly form and redisperse supported, catalytically active Pt clusters through control of its surface morphology and oxidation state. In the present article, we systematically and independently tune these parameters during CeO_2_(111) film synthesis to investigate their influence on the dimensionality (2D vs 3D) and sintering behavior of size-selected Pt_20_ clusters. We present recipes for atomically flat CeO_2_(111) islands and closed films with a thickness of up to 18 monolayers, grown on Rh(111), and characterize them by means of scanning tunneling microscopy (STM), X-ray photoelectron spectroscopy (XPS), X-ray diffraction (XRD), and low-energy electron diffraction (LEED). Remarkably, XRD and LEED reveal an epitaxially grown, crystalline, and relaxed closed film of a single domain, with cube-on-cube alignment. Bulk or exclusive surface reduction is achieved by ultra-high vacuum annealing or room temperature CH_3_OH dosing and annealing cycles, respectively. The methanol procedure forms oxygen vacancies only in the surface without reducing the deeper layers of the film or introducing roughening. From STM images, we extract detailed height distributions and coverages of Pt_20_ clusters and find that Ostwald ripening already sets in around 600 K on both, fully oxidized and surface-reduced ceria, without any indication for cluster diffusion and coalescence. XPS shows that atom detachment during sintering leads to the intermediate formation of Pt^2+^ species on oxidized ceria, in line with the redispersed single atoms at step edges observed in the literature. Strikingly, while the clusters appear similarly upon deposition on both supports, they show a distinct temperature-dependent dimensionality upon annealing: Exclusively 3D clusters form on the oxidized support, while most clusters on the reduced support adopt a flat, 2D geometry upon sintering, stabilized by O vacancies.

## Graphical abstract

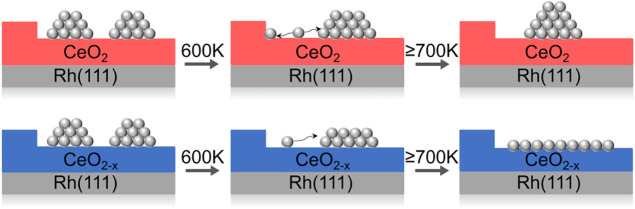

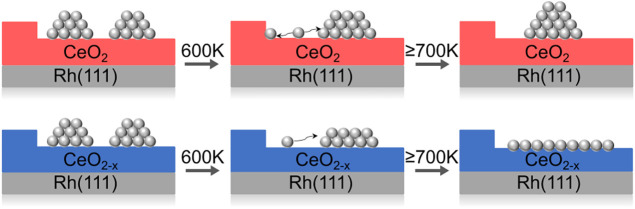

## 1 Introduction

The possible applications of transition metal-decorated ceria reach far beyond the classical automotive three way catalyst,^1,2^ ranging from the water–gas–shift reaction^3,4^ to methanol steam reforming^5,6^ and various organic redox reactions.^7^ All of those applications share a common ground: Ceria is a reducible oxide and acts as an oxygen reservoir due to the high oxygen mobility and facile interconversion between Ce^3+^ and Ce^4+^.^8–10^ As a consequence, ceria can balance charges, and, in its reduced state, it exhibits oxygen vacancies, which represent adsorption and dissociation sites for reactants.^11,12^ In combination with noble metals like Pt, Au, and Pd, it forms an excellent catalytic system for Mars-van-Krevelen-type reactions.^13–15^ Here, the charge state of metallic catalyst particles can be influenced by this highly changeable support, thus tuning activity: This charge transfer can be bidirectional, as shown for supported Rh particles,^16^ depends on the particle size and reaches an intrinsic limit set by the support.^17^ These effects are linked: On the one hand, O vacancies can act as nucleation sites for, e.g., Pt particle formation,^18,19^ while on the other hand, charge transfer from the adsorbed metal may alter the ready availability of lattice oxygen for catalytic applications.^20^

In the subnanometer cluster regime, this complex interdependence is further linked with specific size-dependent catalytic activity, particularly in the non-scalable size regime.^21,22^ During an ongoing chemical reaction, while varying between reducing and oxidizing conditions, such small clusters have been shown to dynamically form and redisperse on extended ceria films^23^ as well as on powder supports.^24,25^ In this redispersion process, the Pt atoms are incorporated as Pt^2+^ species at step edges,^23^ but the catalytically active species in oxidation reactions are the clusters that are neutral,^24^ with some positively charged atoms at the interface to ceria.^17^ A study by Zhou et al. postulated O vacancies as nucleation sites for the clusters.^18^

Bulk ceria with a composition of CeO_2–*x*_ (with 0 ≤ *x* < 0.5) crystallizes in the CaF_2_ structure, where Ce^4+^ cations form a face-centered cubic (fcc) lattice, and each of them is directly surrounded by eight O^2–^ anions.^26,27^ The most stable facet is the (111)-facet, a Tasker type 2 surface^28^ with repeating O–Ce–O trilayers that contain a net zero dipole moment and have a crystallographic height of 3.12 Å.^26,27,29^ Deviations from surface stoichiometry manifest in O vacancies^30^ that can form on all facets, although with structure-dependent formation energies (highest for the (111) facet^10,31,32^ ). Lattice oxygen can, for example, be removed by ultra-high vacuum (UHV) annealing, leading to bulk and surface reduction, or by reduction with CH_3_OH, generating O vacancies exclusively in the surface region.^12^ Each removal of a surface oxygen leads to the localization of its two 2p electrons in empty 4f orbitals of two Ce^4+^ ions in rather close vicinity to the oxygen vacancy, forming small Ce^3+^ polarons that add to the conductivity of ceria via a hopping process, if a thermal barrier is overcome.^33–36^ O vacancies can be localized in the first or the second O layer of the first O–Ce–O trilayer, whereby the lower, subsurface vacancies have been suggested in theoretical work to be more stable by 0.12 eV and, in contrast to an earlier publication,^37^ highly mobile already at room temperature.^38^ Further, O vacancies seem to act as nucleation centers for and stabilize small metal nanoparticles;^18,39^ however, calculations have suggested that they do not act as trapping centers for small Pt_3–6_ clusters, and indeed the cluster binding energy even increases with the distance to an O vacancy.^40^

The epitaxial growth of ceria thin films on noble metal substrates constitutes a simple way of obtaining flat, clean, and bulk-like ceria surfaces and has been demonstrated by several groups, e.g., on Cu(111),^41,42^ Pt(111),^43–45^ Ru(0001),^46^ Si(111),^47^ and Rh(111)^9,29,48,49^ substrates, thus circumventing the difficult preparation of single crystals and proposed related fluorine impurities.^50^ While we are mostly interested in several ML thick films that are representative of bulk ceria, ultimately thin (sub-) ML films, i.e., islands, provide important information about the ceria interaction with the underlying metal substrate: Often, a lattice mismatch leads to Moiré structures^41,51,52^ and the islands tend to be more reduced than thicker films prepared under similar conditions.^9,43,51^ We chose a Rh(111) substrate because it permits high annealing temperatures to tune the CeO_2_(111) film roughness and temperature-induced surface reduction across a wide parameter range. At the same time, a peak overlap between our deposited Pt_20_ clusters and the substrate is avoided in X-ray photoelectron spectroscopy (XPS). In our studies, the ceria films have to fulfill the following requirements: (i) to fully cover the Rh substrate for catalytic studies, (ii) to be crystalline and atomically flat, with reproducible step density for scanning tunneling microscopy (STM) studies of cluster dynamics, and (iii) to be precisely defined in their surface and bulk oxidation state.

In this work, we present recipes for the synthesis of crystalline ceria (111) thin films on Rh(111) with controlled flatness, step density, and oxidation state. Hereby, we increase the number of O vacancies with methanol (CH_3_OH) using only temperatures up to 600 K, thus avoiding simultaneous changes in the surface morphology. In the second part, we investigate the temperature-dependent sintering of size-selected Pt_20_ clusters on atomically flat, oxidized CeO_2_(111) and reduced CeO_1.94_(111) films. We chose this relatively large cluster size to minimize sintering by diffusion and coalescence (Smoluchowski ripening). In a combined STM and XPS study, we show that the dimensionality of sintered clusters changes depending on the support oxidation state. In particular, we investigate the Pt oxidation observed upon atom detachment during Ostwald ripening.

## 2 Experimental Methods

All experiments have been performed in a UHV chamber (base pressure <2 × 10^–10^ mbar) directly connected to the STM chamber (base pressure <3 × 10^–11^ mbar), which is separated by a gate valve. Care has been taken to precisely control the sample temperature: A pyrolytic boron nitride heater and a type K thermocouple, both in direct contact with the sample, are mounted on the sample holder and ensure highly reproducible heating conditions; the absolute temperature values are accurate to ±5 K. Purity of sample preparation gases was regularly checked with a quadrupole mass spectrometer.

The Rh(111) substrate (Surface Preparation Laboratory) was cleaned by multiple cycles of Ar^+^ ion sputtering (4 × 10^–5^ mbar Ar, 1.5 kV, 10 min), followed by three annealing cycles between 773 and 1223 K in 5 × 10^–7^ mbar O_2_ to alternatingly segregate carbon impurities from the bulk (1223 K) and react them off (773 K). This procedure was followed by another constant-temperature annealing step in oxygen (5 × 10^–7^ mbar O_2_, 773 K, 30 min). The cleanliness of the substrate was checked regularly by XPS.

Cerium (NovaElements, 99.95%) was evaporated onto the Rh(111) substrate at room temperature from a home-built ribbon evaporator (original design by the group of Peter Feulner, TUM)^53^ in 3 × 10^–7^ mbar O_2_. To obtain reproducible film thickness, the deposition was monitored by a quartz crystal microbalance, mounted on the opposite side of the evaporator filament, and thus continuously measuring the evaporation rate (typically 0.5–1 ML/min). All indicated thickness values, however, are determined by the attenuated Rh 3d signal from XPS measurements, as described below. Note that we define a monolayer as one complete O–Ce–O trilayer. The deposition step was followed by alternating oxidizing and reducing treatments, as described below. The cleanliness of the films was checked by XPS. In surface-sensitive measurements, i.e., grazing emission combined with the Mg Kα source, we observe a ubiquitous, small Al impurity which originates from the cerium evaporation process and was also observed in ref 23 (Supporting Information), where it was claimed not to interfere with the Pt/CeO_2_ system. Such Al and also Si impurities are generally observed at grain boundaries, as discussed in ref 54.

Size-selected Pt clusters were deposited using a laser-ablation cluster source,^55^ which uses the second harmonic emission of a Nd/YAG laser to evaporate Pt from a rotating target, thereby forming a plasma. Subsequently, this plasma is cooled in the adiabatic expansion of a He pulse (Westfalen AG, grade 6.0), leading to the condensation of Pt clusters of a broad size range. The formed supersonic cluster beam is guided through a set of electrostatic lenses, a 90° bender selects only positively charged cluster ions, and finally a quadrupole mass filter selects the desired cluster size with atomic precision. Deposition of the clusters onto the support occurs under soft-landing conditions (<1 eV/atom), which is achieved by applying a retarding field to the sample.

STM measurements were performed with a commercial VT-AFM instrument (Scienta Omicron) at room temperature (RT) in constant current mode using either commercially available Pt/Ir tips (Unisoku) or home-etched W tips; we checked that the tip material had no influence on contrast and apparent cluster height. The images were then quantitatively analyzed with respect to support morphology, density of steps and defects, and cluster apparent heights and coverage. Coverages are indicated in the number of clusters per unit area. For the systematic cluster sintering investigation, the sample was annealed in UHV in 100 K steps and held at the maximum temperature for 10 min each, and subsequently, STM images were taken after each annealing step at room temperature. Images were processed with the software Gwyddion,^56^ using the plane subtraction, row-by-row alignment, and plane leveling tools as a standard routine. Cluster height and coverage analyses were performed using a home-written Igor routine, where clusters are detected by an intensity threshold and their height is determined terrace by terrace with respect to the median of the highest of 20% pixels around each cluster. Since the definition of the background is of major importance and can influence the apparent cluster height distribution, a comparison of different background subtraction methods is shown in Figure S1. For each measurement condition, three large-scale images were used for quantitative analysis and combined with histograms of the cluster height distribution. The step edge length was determined using the length-calibrated drawing tool in ImageJ.^57^

XPS spectra were measured in the same UHV setup with a combined Al Kα and Mg Kα X-ray source (SPECS, XR 50) and a hemispherical analyzer (Omicron, EA 125) in two configurations: (i) “bulk”-sensitive, measured in normal emission with Al Kα radiation, and (ii) surface-sensitive, measured in grazing emission at 70° to the surface normal using Mg Kα radiation. For data evaluation and peak fitting, the software KolXPD^58^ was used. For all fitted Pt 4f spectra, we used slightly adapted fit parameters from ref 59: In our case we added a Shirley background to allow integration of the peaks and fixed the binding energy difference between the Pt^0^ and Pt^2+^ species to 1.9 eV. As we will show below, XRD shows that the film thickness varies across the sample. We determine average film thickness values from our XPS (3 mm spot size) using the simple exponential attenuation dependence of the inelastic mean free path (IMFP) method. Here, we use attenuation of the Rh 3d level by the ceria film measured in normal emission. The IMFP of 21.2 Å was calculated using the TPP-2M method of the NIST inelastic mean free path database^60^ with 16 valence electrons (for a fully stoichiometric CeO_2_ film), a band gap of 6 eV^61–63^ and a CeO_2_ density of 7.22 g/cm^3^.^64^ To deduce the number of deposited monolayers (ML), we used the literature value of 3.12 Å, which corresponds to the thickness of one O–Ce–O trilayer.^27,29^ We thus define 1 ML as one such trilayer. The quantification of the ceria oxidation state and the peak assignment of the Ce 3d spectra are described in detail in Supporting Information Section S2. Note that we can only determine the precise degree of reduction by XPS and thus assume the oxidation state to be similar in STM experiments on films that are prepared under identical conditions. For temperature-programmed desorption (TPD) measurements, we use our so-called sniffer-MS setup, which is described in detail elsewhere.^65,66^ To perform this kind of experiment, we cooled the sample down to 200 K, where we dosed 15 L of isotopically labeled C^18^O to differentiate the TPD signal from the background CO. The samples were heated with a linear temperature ramp of 1 K/s up to a temperature of 800 K, while measuring the *m*/*z* = 30 trace (next to *m*/*z* = 28, 44, 46) in the quadrupole mass spectrometer (QMS). Low-energy electron diffraction (LEED) measurements were performed with a commercial instrument (SPECS, ErLeed) in combination with an SLR camera in the same chamber.

X-ray diffraction (XRD) measurements were performed on a 6-circle diffractometer in combination with a microfocus Cu lab source at the DESY Nanolab.^67^ The X-rays were monochromatized using parabolic gradient multilayer optics. The diffraction experiments were performed in grazing-incidence geometry to maximize the signal-to-noise ratio of the thin film X-ray scattering with the incident angle fixed at the critical angle for total external reflection of Rh (0.48°). For the grazing incidence X-ray diffraction experiment, surface coordinates were used: *a*_s_ = *b*_s_ = *a*_0_/ $\sqrt{2}$ , c_s_ = *a*_0_ $\sqrt{3}$ , α = β = 90°, γ = 120°, with *a*_0_ = 3.80 Å, the bulk lattice constant of the fcc Rh lattice. XRD momentum transfers *Q* are given in relative reciprocal lattice units (r.l.u.) *H*, *K*, and *L* with *Q* = *H* **a**_s_* + *K* **b**_s_* + *L* **c**_s_* and the surface reciprocal lattice vectors **a**_s_*, **b**_s_*, and **c**_s_*. The axes *a*_s_ and *b*_s_ lie in the surface plane, while c_s_ is perpendicular to it. The first Bragg peak in the specular direction is labeled as (003), which corresponds to (111) when using the bulk fcc unit cell.

## 3 Results and Discussion

In the following, we start off by presenting a thin film growth strategy that allows the tuning of ceria (111) step edge density and surface oxidation state before presenting first data on the sintering behavior of Pt on two differently reduced ceria surfaces.

### 3.1 Tuning and Characterizing Highly Defined CeO_2–*x*_(111) Films

#### 3.1.1 Atomically Flat CeO_2–*x*_(111) Islands on Rh(111)

Before growing closed, several ML thick ceria films, we start with atomically thin ones to understand in detail the ceria–Rh interface. Figure 1 shows the characterization of ceria islands on the Rh(111) surface, which have been synthesized building on successful recipes on transition metal substrates.^68,69^ To avoid the possible formation of alloys with Rh, we evaporate Ce in 1 × 10^–7^ mbar O_2_ at room temperature, followed by annealing to 973 K for 15 min in the same O_2_ atmosphere. Such ultrathin films can be imaged by STM particularly well, even at low bias voltages.^70^ Here, unoccupied states are imaged preferentially, with the apparent protrusions at the atomic scale centered on Ce atoms.^38,52^ This contrast is the one generally observed when scanning at room temperature, i.e., significantly below temperatures where conductivity by polaron hopping dominates—as is the case throughout this article.

**Figure 1:**
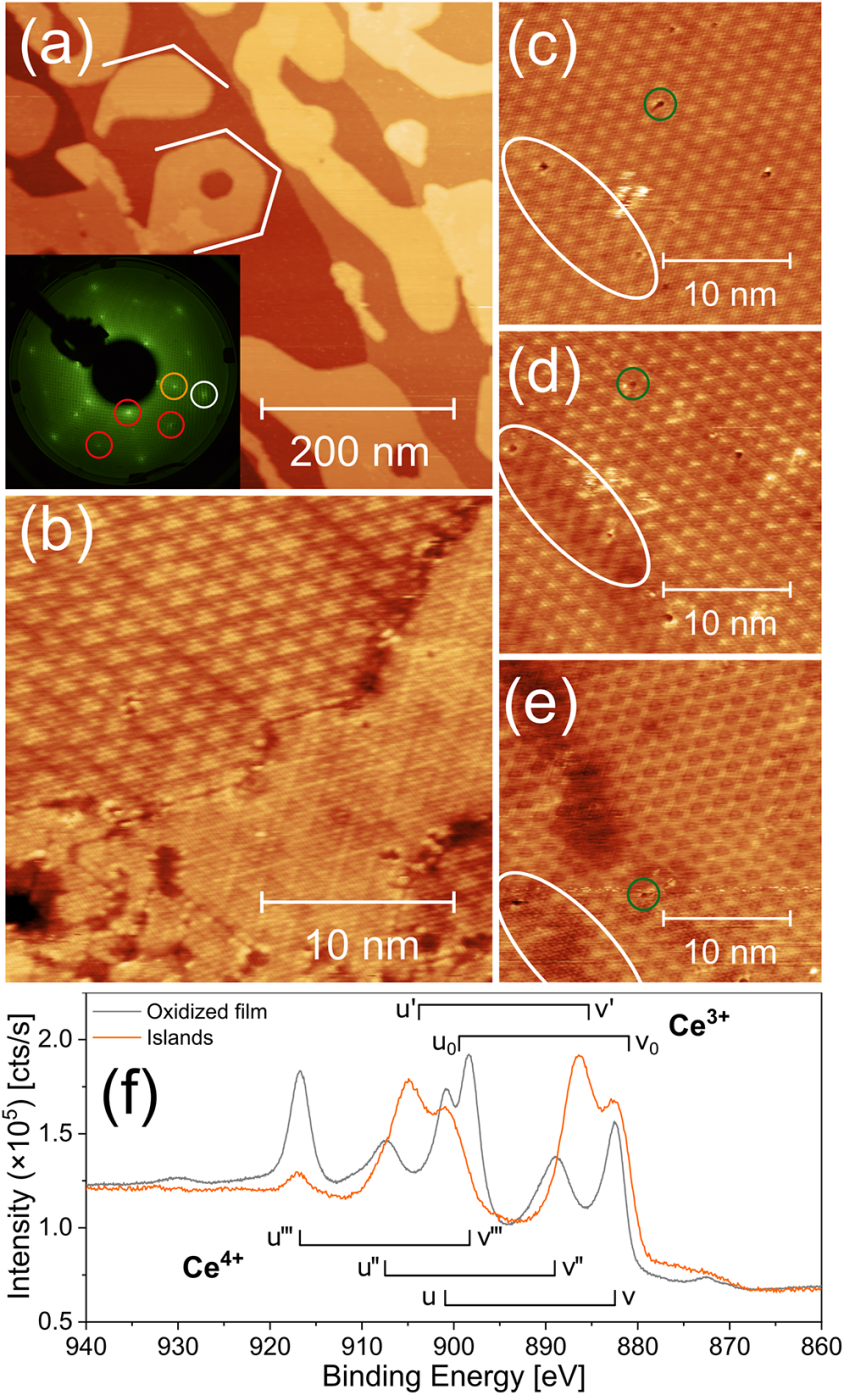
(a–e) STM images of CeO_1.58_ islands on Rh(111), prepared by Ce evaporation in 1 × 10^–7^ mbar O_2_, followed by annealing to 973 K in the same atmosphere. (a) Bright areas in a large-scale STM image are the islands, and darker areas are the Rh terraces. Inset: LEED image of this surface, taken at 91 eV beam energy. The white circle marks the Rh spots, the orange circle represents the CeO_2_ (1.4 × 1.4) superstructure, and the red circles show the O(2 × 1) superstructure, which forms on the bare Rh terraces. (b) STM image of an area with two different surface superstructures (Moiré structure [upper left] and “cartwheel” structure [lower right]). (c–e) STM bias series over the same region (with always the same defect marked with green circles as a guide to the eye). The area marked with the white oval shape shows a domain boundary in the Moiré structure. Imaging conditions: (a) *U*_b_ = 1.5 V, *I*_t_ = 300 pA, (b) *U*_b_ = 1.5 V, *I*_t_ = 500 pA, (c) *I*_t_ = 300 pA, *U*_b_ = 2.0 V, (d) *I*_t_ = 300 pA, *U*_b_ = 1.5 V, and (e) *I*_t_ = 300 pA, *U*_b_ = 1.0 V. (f) Ce 3d XPS spectra, taken with Al Kα radiation under normal emission. The orange spectrum corresponds to the STM images; the gray curve shows the spectrum of a 14 ML thick, stoichiometric film (0.7% Ce^3+^), multiplied by a factor of 0.26 to match the background intensities.

The large-scale STM image in Figure 1a includes four Rh terraces (darker areas) covered with approximately 0.5 ML ceria islands (brighter areas). These islands form relatively round shapes and occasionally exhibit approximately hexagonally oriented step edges (as marked by the white lines), which coincide with the orientation of the hexagonal ceria lattice, as can be seen by the atomic resolution images in Figure 1b–e. We observe an apparent island height of 4.2 ± 0.4 Å, which is higher than the 3.12 Å crystallographic thickness of one ideal, stoichiometric O–Ce–O trilayer (for example, line profiles across islands, see Figure S3).^42,71^ The discrepancy becomes even bigger when we consider previous reports in the literature stating that the first 1–2 trilayers on various metal substrates appear even lower, since they are slightly reduced.^41,72,73^ Indeed, the Ce 3d XPS spectrum in Figure 1f reveals a strongly reduced state of the film, corresponding to a stoichiometry of CeO_1.58_. For comparison, we added the Ce 3d spectrum of a 14 ML thick, stoichiometric CeO_2_ film (0.7% Ce^3+^; fit see Figure S1), shown by the gray dashed line, and assigned the peaks according to the well-established nomenclature: The doublets *u* + *v*, *u*″ + *v*″, and *u*‴ + *v*‴ correspond to Ce^4+^, while *u*_0_ + *v*_0_ and *u*′ + *v*′ arise from the Ce^3+^ state, where the *u* peaks correspond to Ce 3d_3/2_ and the *v* peaks to Ce 3d_5/2_.^74–76^ The measured data are thus consistent with ceria islands that are double trilayers.

The LEED image in the inset of Figure 1a shows that these islands are highly crystalline. Since the ceria covers the substrate only partially, we observe three distinct patterns: The white circle marks the Rh­(0,1) spot, the orange circle represents the CeO_1.58_(0,1) spot, which is similar to the well-known $\left(\sqrt{2}\times \sqrt{2}\right)$ superstructure of CeO_2_,^9,29^ and the red circles correspond to the O(2 × 1) superstructure on the bare Rh surface.^77^ With the right contrast, the O(2 × 1) superstructure is also visible in STM images as regions of parallel lines, with domains rotated by 60° (Figure S4).^78^ From the relatively sharp LEED spots, we conclude that both the ceria film and the underlying Rh(111) surface are well-ordered and epitaxially oriented. Figure 1b reveals the concomitant existence of two superstructures in the lattice of the ceria islands. In the upper left part of the image, we observe a highly regular Moiré structure consisting of triangular protrusions, while in the lower right part a “cartwheel” structure dominates, depending on the orientation of CeO_1.58_ islands and the Rh substrate.^52,79^ The atomic resolution in the Moiré structure indicates a buckled (5 × 5) superstructure, in agreement with a report by Chan and Yuhara for a ceria coverage of 0.8 ML on Rh(111).^29^ The contrast within the Moiré unit cell changes for different bias voltages, from round (2.0 V, Figure 1c) and triangular protrusions (1.5 V, Figure 1d) to a honeycomb-like structure (1.0 V, Figure 1e). As the contrast in Figure 1e changes significantly after the first third of the image, we conclude that the contrast is not only bias-dependent but also depends strongly on the composition of the foremost atoms of the tip (as is also evident when comparing Figure 1b,d, recorded at the same bias). In all three images in Figure 1c–e, we observe a domain boundary (marked by the white ellipse), where the Moiré structure is shifted by half a (5 × 5) unit cell. While this domain boundary appears only as shifted protrusions in Figure 1c, the whole area around the domain boundary appears darker in Figure 1d, which is probably due to an electronic effect at the interface. In addition, images in Figure 1c–e also contain several dark defects (one of which is marked with a green circle).

The incomplete covering of the Rh substrate, combined with the strong reduction and electronic effects, does not make these ceria islands themselves ideal supports for supported cluster investigations. That being said, although strained, the large extension of the islands gives an indication that thicker, single-crystalline ceria films can be grown across the entire substrate.

#### 3.1.2 Atomically Flat, Closed CeO_2_(111) Films on Rh(111) with a Defined Oxidation State

In this section, we present a closed, many ML thick flat ceria film, which is suitable as a support for metal nanoparticles and size-selected clusters and permits the use of not only local techniques like STM but also ensemble-averaging methods like XPS or reactivity measurements. Above ∼6 ML thickness, we expect the outermost ceria layer to have bulk-like properties^80^ and image charge effects induced in the Rh substrate not to play an important role. Figure 2 illustrates the different oxidation, reduction, and reoxidation steps of the preparation process of a closed and atomically flat 13 ML thick ceria film, which we will now describe in turn.

**Figure 2:**
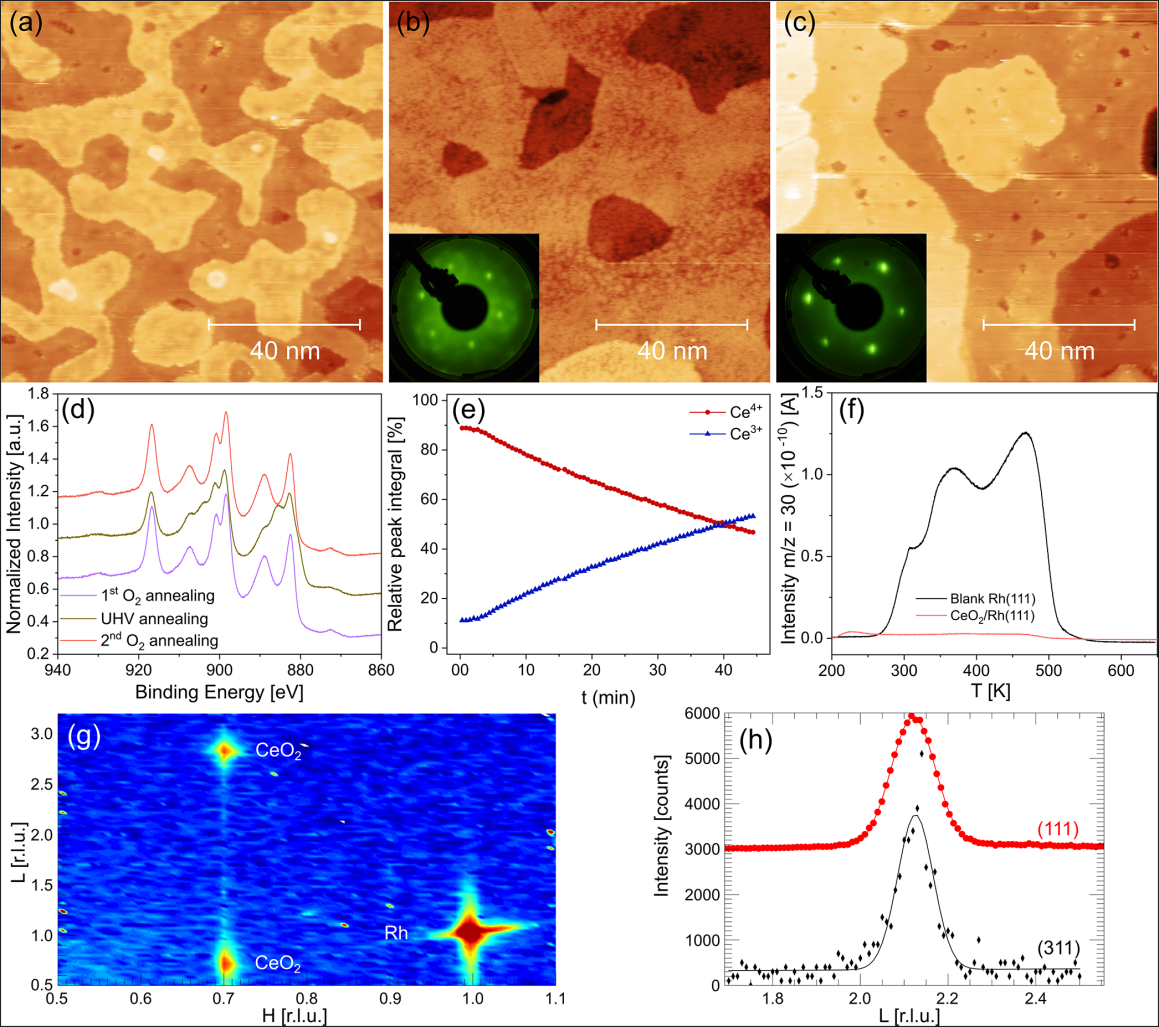
Characterization of a 13 ML ceria film on Rh(111) at different steps during the preparation process. (a–c) STM images of (a) a stoichiometric CeO_2_(111) film after the first round of annealing at 1073 K in 5 × 10^–6^ mbar O_2_ for 15 min, (b) the same film after 45 min of UHV annealing at 1073 K, and (c) after the second O_2_-annealing as in (a). Imaging conditions: *I*_t_ = 300 pA, (a,c) *U*_b_ = 3.5 V, (b) U_b_ = 3.0 V. Insets in (b) and (c) show LEED patterns of the respective films, acquired at 81 eV beam energy. (d) Ce 3d region in XPS after the three preparation steps, taken with Al Kα radiation under normal emission. (e) Time evolution of the relative peak integrals for Ce^3+^ and Ce^4+^ during the 1073 K UHV annealing step, measured with a time resolution of ∼ 35 s per spectrum. (f) C^18^O TPD measurement from the bare Rh(111) surface (black) and the CeO_2_(111) film presented in (c) (orange), both measured with a 1 K/s heating rate. (g) Reciprocal space map in the *H*0*L*-plane of a CeO_2_ film on a Rh(111) substrate. The H and L reciprocal space parameters relate to the Rh substrate using the surface unit cell, see methods section. Small isolated dots are intensity spikes, most likely from cosmic rays. (h) L-scans through two CeO_2_ reflections. The top peak is displaced along the *y*-axis for clarity. The solid lines are Gaussian fits to the profiles. From their width the crystalline film thickness can be determined.

First, the STM image in Figure 2a shows the film after Ce evaporation in 3 × 10^–7^ mbar O_2_ at room temperature and a 15 min annealing step at 1073 K in 5 × 10^–6^ mbar O_2_, to flatten and order the film. At the annealed stage in (a), the film is already relatively flat, exhibiting only monatomic islands with some of their edges oriented along hexagonal directions, as also observed for the islands in Figure 1a. The Ce 3d XPS region in Figure 2d (purple) shows that the film is nearly stoichiometric with a fit-derived Ce^3+^ content of 3.1%.

Second, if the same film is annealed to 1073 K in UHV for 45 min, the islands spread to a more homogeneous film with extended terraces, as shown in Figure 2b. This, at first sight, seems advantageous for our model catalyst studies. However, the surface now shows an apparent roughening within the flat terraces, which coincides with a particular reconstruction in the LEED pattern (inset). In comparison to the islands in the previous section, we do not observe spots from the Rh(111) substrate anymore. This is in line with an IMFP of the electrons of only ∼5 Å at 81 eV beam energy (estimated with the NIST IMFP calculator^60^), which corresponds approximately to the first two CeO_2_ trilayers. In the LEED pattern, this reconstruction appears as a circular fine structure around the hexagonally arranged Ce spots, and we measure a (2.7 × 2.7) periodicity. However, since these LEED spots are rotationally smeared out, we cannot determine an angle with respect to the Ce spots. The observed structure thus fits well with two previously reported structures: Wilkens et al. and Duchoň et al. observed a $\left(\sqrt{7}\times \sqrt{7}\right)R19.1^{{}^{\circ}}$ structure for CeO_2–*x*_(111) films on Si(111) and Cu(111), with a Ce^3+^ concentration of 57 and 65%, respectively.^81,82^ Both groups assign their reconstruction to an ordered structure of O vacancies upon the formation of an ordered, so-called *ı*-phase, Ce_7_O_12_. Furthermore, (3 × 3) domains could be present, also observed on partially reduced ceria films.^43,82^ The corresponding XPS spectrum in (d) (green) exhibits a significant reduction of the film, manifest by a Ce^3+^ concentration of 43% that indeed corresponds to the CeO_1.7_ stoichiometry of Ce_7_O_12_, i.e., the structure with ordered O vacancies. As reported previously,^82^ the surface is highly interactive with the STM tip in this reduced state, which makes it difficult to obtain atomic resolution. However, in between large, streaky and cloudy features, we occasionally observe areas of regularly ordered, 3-fold depressions (see Figure S5), having a similar appearance to the O vacancies mentioned before. The structural models given by Duchoň et al. for the reduced structures on Cu(111) contain both, surface and subsurface vacancies—we can thus assign the observed depressions in our ceria film on Rh(111) to both species. To observe the reduction step in more detail, we performed time-resolved XPS measurements of the Ce 3d region during 45 min of UHV annealing at 1073 K. The linear evolution of the relative peak ratios for Ce^3+^ and Ce^4+^ in Figure 2e suggests that the reduction of the CeO_2_ film at this temperature is an inherently slow, continuous process. Note that the reduction already starts during the ramp to the annealing temperature and that the curve therefore already starts at ∼10% relative peak integral. Further, it is interesting that a complete reduction to Ce_2_O_3_ at this temperature could not be obtained (data not shown), which could suggest that the O loss that starts from the surface does not fully propagate down to deeper layers, resulting in a depth-dependent gradient in the number of Ce^3+^ ions.

If the film is now reoxidized in a third step, under the same conditions as during the first O_2_-annealing step, the roughening within the flat terraces is lifted, further confirming the assignment of the depressions to O vacancies (Figure 2c). Remarkably, the terraces remain as extended as after the reduction step, and the step density is significantly reduced in comparison to the first oxidative annealing step. According to the corresponding Ce 3d XPS spectrum (Figure 2d, coral color), the film became reoxidized back to a Ce^3+^ content of 3.0%, and also the LEED reveals that the ordered structure of O vacancies is gone and only the spots from the ceria lattice remain (inset to Figure 2c). Importantly, one can clearly see that the diffraction pattern symmetry at this electron energy is distinctly 3-fold and that hence the highly flat and nearly stoichiometric CeO_2_ film obtained by this recipe has a single orientation (see Figure S6 for energy-dependent LEED images). Indeed, in these several ML thick films, we do not observe any domain boundaries in our STM images, as, for example, seen clearly in ceria films on Ru(0001) by Nilius et al.^83^

XRD measurements confirm that the bulk of the film is indeed single crystalline. Figure 2g shows a reciprocal space map of a similarly prepared film in the *H*0*L* plane. It shows one reflection from the Rh substrate and two peaks from the CeO_2_ film. The strongest reflection at (1,0,1) belongs to the Rh. The peaks at (0.7,0,0.7) and (0.7,0,2.8) are indexed in the figure using the Rh surface unit cell as a reference. They belong to the CeO_2_ film and correspond to the CeO_2_ bulk (1̅11) and (202) reflections. The faint line of intensity along L connecting the two film reflections is the film crystal truncation rod signal, in line with an atomically smooth surface of the CeO_2_ film (in agreement with the STM images). A similar signal is seen along L around the Rh reflection, but it extends less far from the Bragg peak, pointing toward an enhanced interfacial roughness. The streak along H is due to the X-ray source’s dispersion. In a rocking scan (not shown), the film reflections do not show any signs of enhanced mosaicity (around 0.2°, which is close to the X-ray beam angular divergence), indicating that the thin film is single crystalline. The fact that the CeO_2_ peaks are found in the Rh plane shows that the ceria lattice is aligned cube on cube on the Rh substrate along the [111] direction, with alignment of the corresponding in-plane axis. Furthermore, the absence of twinned peaks along the [111] direction (which we would expect at (0.7,0,1.4) in the figure) indicates that there is only a single domain, in accordance with the LEED observations. From the position of several Bragg peaks, the film is found to be fully relaxed cubic with a lattice parameter of a_cub_ = 5.40 ± 0.02 Å. The obtained CeO_2_ film cell parameter corresponds within the error bar very well to that of bulk stoichiometric ceria^84^ and thus an interfacial coincidence on specific lattice sites is not expected. Figure 2h shows scans along the *L*-direction through two different CeO_2_ reflections. Shown are the (0,0,2.1) reflection in the specular direction (red circles, top) and the (1.4,-0.7,2.1) reflection (black asterisks, bottom), which in bulk coordinates correspond to the (111) and (311) reflections, respectively. The intensity profiles are fitted using a Gaussian. The width of these peaks is inversely proportional to the crystalline thickness *t* as *t* = *c*_s_/Δ*L*. Both peaks in Figure 2h have a width of approximately Δ*L* = 0.1 r.l.u. Measurements of the specular peak on different parts of the sample showed a scatter in the peak widths in the range of Δ*L* = 0.1–0.3 r.l.u. From these values, it follows that the thickness is not homogeneous over the whole sample and varies in the range *t* = 2–7 nm (on average corresponding to the nominal thickness of 13 ML, ∼4.0 nm, determined by XPS).

In the last characterization step, we rule out the influence of the underlying Rh(111) substrate on catalytic activity measurements by checking whether the film is fully closed. To this purpose, we compare C^18^O TPD spectra of the bare, freshly cleaned Rh(111) surface and the reoxidized ceria film (Figure 2f). The two curves have been measured under identical experimental conditions and with the same C^18^O exposure (150 gas pulses, corresponding to approximately 15 L). Visual inspection shows no significant desorption from the ceria-covered Rh(111) sample. Integrating the peaks gives a <4% area for the ceria film sample compared to the bare Rh(111), which includes signals from defects, exposed Rh, and background effects, thus confirming that the film is essentially fully closed. Also the STM images in Figure 2c do not clearly show uncovered areas. Yet, when looking at larger-scale images, it becomes evident that the film occasionally exhibits deeper holes and even thin, hexagonally connected cracks, which could be deep enough to expose the substrate (Figure S7). Nevertheless, at the typical cluster coverages that we use for TPD and activity measurements, the influence of Rh(111) is negligible. We thus conclude, that the described sequence of oxidation, reduction and reoxidation provides atomically flat and essentially fully closed CeO_2_(111) thin films, which can be used as an ideal support for deposition of size-selected clusters in catalytic model systems.

#### 3.1.3 Surface-Preferred Reduction with CH_3_OH

In the previous section, we discussed a method for reducing ceria thin films by high-temperature annealing in UHV. However, this step induces a strong rearrangement of the whole film, roughness within the terraces, and potentially an unknown vertical gradient of Ce^3+^ ions. To get better control over the surface reduction of the film and investigate its influence on sintering and structural changes of small metal clusters deposited thereon, we now apply a second method, as applied previously in the literature.^12,85–87^ Hereby, the previously prepared near-stoichiometric film is gently reduced exclusively in the topmost trilayer by exposure to methanol and annealing to comparatively low temperatures, i.e., 600 K. On CeO_2_(111), CH_3_OH can adsorb on stoichiometric surfaces and, dissociatively, at O vacancies, leading to the formation of surface methoxy and hydroxyl species, which subsequently can either recombine to CH_3_OH or react towards H_2_O, thus creating more vacancies.^87^ In TPD measurements with isotopically labeled Ce^18^O_2_(111), Mullins et al. showed that this reduction of the ceria surface already occurs at around 200 K, forming H_2_O, while formaldehyde and methanol only desorb at 560 K.^85^ The authors state that until that temperature, the active sites for the reaction are blocked by the remaining methoxy species, while introducing additional O vacancies increases the reduction rate due to overall more available reaction sites. In our reduction approach, we utilize this mechanism by applying a cyclic CH_3_OH treatment of the CeO_2_(111) film to obtain a significant but still limited number of O vacancies in the top trilayer, without reducing deeper layers. Figure 3a shows an atomic-resolution STM image of a 4.7 ML oxidized CeO_2_(111) film, which was prepared by the method described in Section 3.1.2. Note that the STM images in Figure 3 are drift-corrected by shearing to obtain the regular hexagonal lattice, in a process we have described in ref 88.

**Figure 3:**
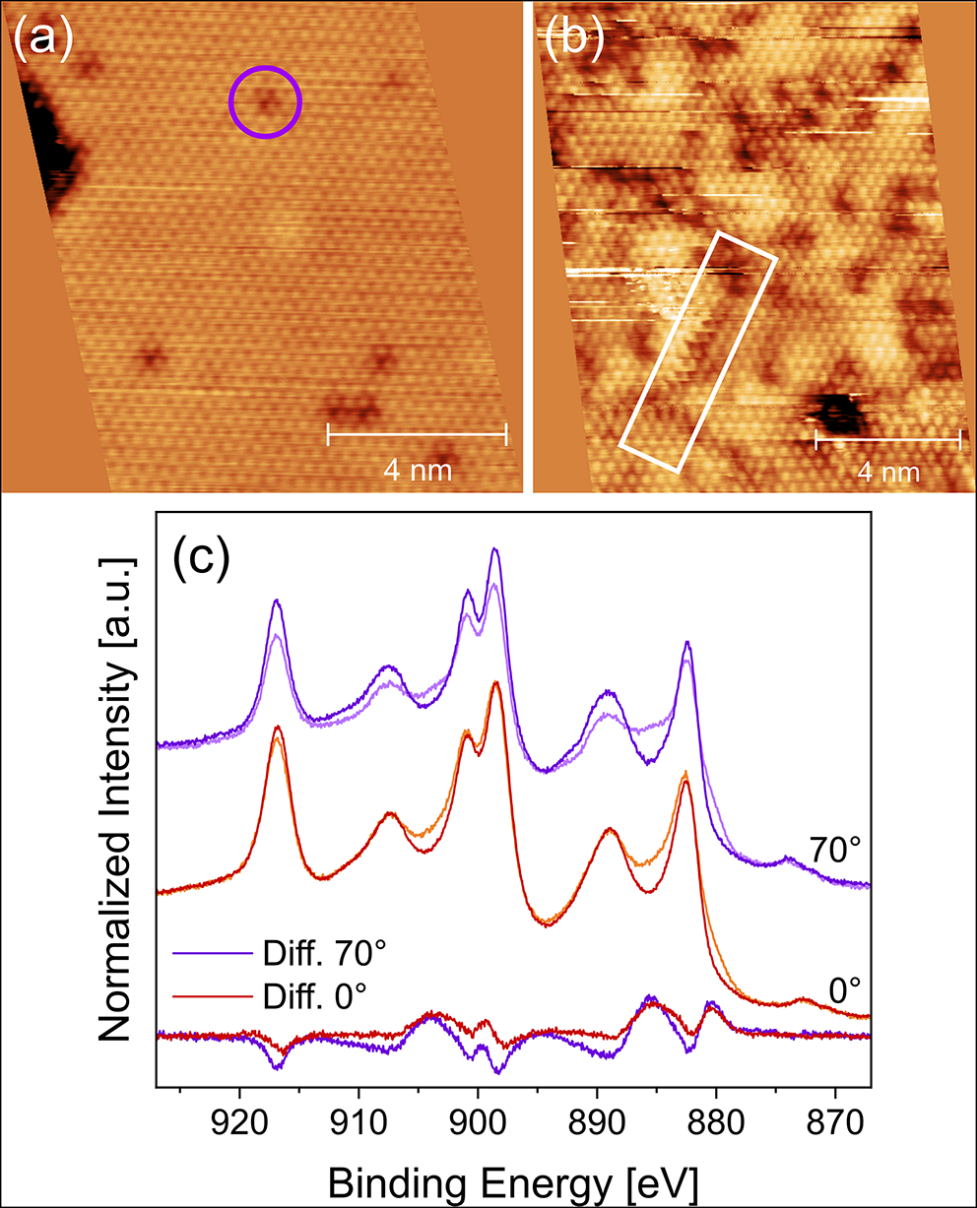
Surface reduction by methanol treatment. (a) Atomic-resolution STM image of a 4.7 ML thick ceria film. A point defect is marked by a purple circle. (b) The same surface after two rounds of dosing 10 L CH_3_OH at room temperature and annealing to 600 K for 5 min with a heating ramp of 1 K/s exhibits a drastically larger number of defects. The white rectangle marks an area where the vacancies form a chain-like structure oriented along the Ce lattice. Imaging conditions: (a) *U*_b_ = 2.0 V, *I*_t_ = 2.5 nA, (b) *U*_b_ = 4.5 V, *I*_t_ = 1.2 nA. (c) Comparison of the Ce 3d XPS region of an oxidized (darker shades), 7.1 ML thick film and the CH_3_OH-reduced film from (b) (lighter shades) measured in grazing (purple shades) and normal emission (red/orange shades). The spectra are normalized for clarity. The difference between the two respective pairs of spectra is shown below.

Starting from the reoxidation step of the thin film synthesis, the film already contains a low number of dark point defects, one of which is marked with a purple circle. These are well-known and commonly interpreted as O vacancies.^43^ Their appearance in these highly resolving STM images closely resembles the subsurface O vacancies simulated by Wolf et al. with the 3-fold symmetry from rotational averaging taking into account electron hopping on time scales faster than that of an STM measurement.^38^ However, in contrast to the high vacancy mobility proposed by these authors (as discussed in the Introduction), the defects observed in our STM images are immobile at RT. We do not observe the surface vacancies suggested in the same paper, which might be due to their high mobility. In general, several recent theoretical studies have focused on the O vacancy dynamics,^33,35,36,38^ but experimental evidence is still missing. After the film synthesis, we dosed 10 L of CH_3_OH (1.3 × 10^–7^ mbar for 100 s) onto the surface at room temperature, followed by a 5 min annealing step at 600 K in UHV with a 1 K/s heating ramp to desorb the remaining adsorbed species. This process is repeated a second time, yielding a strongly increased number of O vacancies, as can be seen in Figure 3b. In some areas, like the one marked with a white rectangle, they appear to be clustered together, forming chain-like structures along the visible Ce lattice, which has been observed before for several examples of CeO_2–*x*_(111) surfaces.^37,89^ Figure 3c shows the Ce 3d region of a stoichiometric, similarly prepared but slightly thicker film (7.1 ML) and the Ce 3d region corresponding to the film in Figure 3b, measured in normal (0°, red, and orange) and grazing emission (70°, dark and light purple). Based on the IMFP, calculated for kinetic energies that take into account an average binding energy of 900 eV for the Ce 3d region, the respective radiation energies and simple geometric considerations, we can estimate that 63% of the “bulk”-sensitive signal in normal emission spectra (corresponding to a signal decay to 1/e) originate from the upper 4.1 ML and 63% of the surface-sensitive signal in grazing emission spectra from the topmost layer (1.0 ML). Indeed, the “bulk”-sensitive spectra before (red) and after the reduction treatment (orange) change significantly less, as manifest in the flatter difference spectrum at the bottom of the image (red). At the same time, the surface sensitive spectra before (dark purple) and after the reduction (light purple) indicate a pronounced reduction, even more evident in the corresponding difference spectrum (purple) with strong negative Ce^4+^ peaks and positive Ce^3+^ peaks. Thus, it can be concluded that the reduction is mostly taking place in the topmost layer of the film. From the grazing emission spectra, the amount of Ce^3+^ in the outermost layer of the reduced film is determined to be 39%. Large-scale images of the film before and after the reduction treatment confirm that the overall long-range structure is not influenced by this reduction method, as shown in Figure S8.

### 3.2 Temperature-Dependent Morphology and Sintering of Pt_20_ on Oxidized and Reduced CeO_2_(111)

Having developed two recipes for the preparation of equally flat ceria thin films with different degrees of surface reduction, we now deposit size-selected Pt_20_ clusters on these oxidized and surface-reduced CeO_2_(111) films and investigate the temperature-dependent cluster morphology and sintering. From grazing emission XPS, we determine a Ce^3+^ content in the first trilayer of 1–3% for the oxidized (≙ CeO_2_) and 12% for the reduced sample (≙ CeO_1.94_), corresponding to ∼2 and 6% of the O vacancies, respectively. The number of deposited Pt_20_ clusters (∼0.03 clusters/nm^2^) is 0.4% cluster ML with respect to the number of surface Ce atoms, respectively 8% Pt atom ML (numbers that are directly comparable to the O vacancy coverages).

We annealed the as-prepared samples sequentially in steps of 100 K, keeping the sample at the maximum temperature for 10 min each, and after each temperature step, we measured STM and XPS at room temperature in two separate but identical measurement series. Figure 4 shows representative STM images after each annealing step for the oxidized (top row) and reduced samples (second row). The STM data are further analyzed in terms of cluster coverage (third row) and in terms of apparent cluster height (Figure 5), which we determined for all Pt_20_ clusters not located directly at the step edges. Here, dashed vertical lines indicate the atomic layers of a Pt(111) single crystal (i.e., 2.25 Å) as a reference and guide to the eye. The corresponding Pt 4f XPS spectra, shown in Figure S9, are fitted with two doublets at a distance of 1.9 eV, which we assign to Pt^0^ and Pt^2+^.^59^ The respective peak integrals are plotted in Figure 4r as a function of annealing temperature, while the binding energy (BE) of the Pt^0^ component is given in Figure 4s.

**Figure 4:**
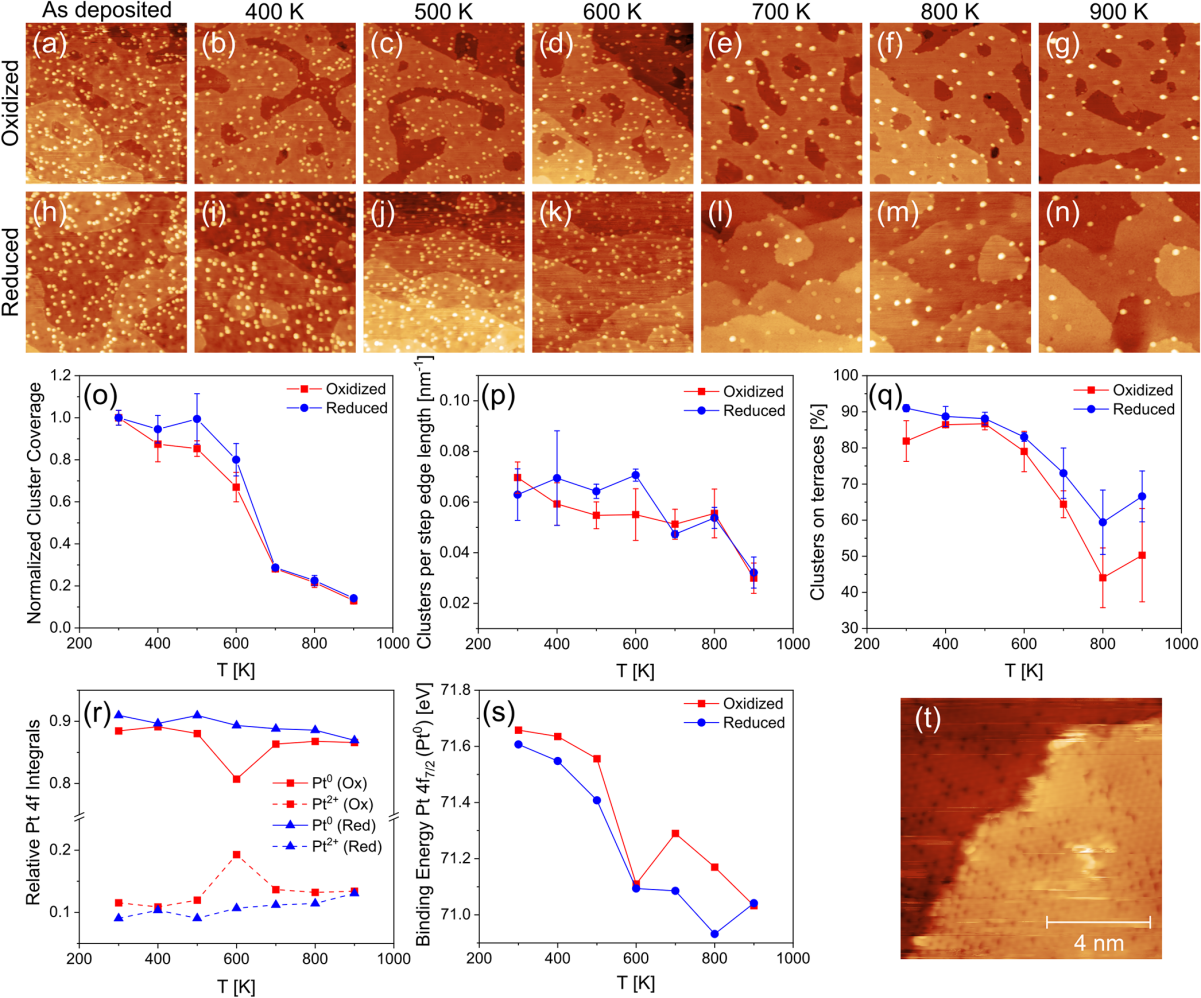
(a–n) 100 × 100 nm^2^ room temperature STM images of (a,h), as deposited and (b–g, i–n) sintered Pt_20_ clusters (approximately 0.03 clusters/nm^2^) on oxidized CeO_2_(111) (12 ML, top row) and reduced CeO_1.94_(111) (18 ML, second row) thick films. Each image was taken after cooling down from a 10 min annealing period at the respective temperature. All images are set to a similar contrast scale so cluster brightness, which is proportional to their apparent height, can be compared directly. Imaging conditions: (a–g) *U*_b_ = 3.5 V, *I*_t_ = 200 pA, (h–n) *U*_b_ = 3.0 V, *I*_t_ = 200 pA. From these and further STM images, we extract (o) the cluster coverage, which we normalize to the initially deposited number of clusters per unit area, (p) number of clusters per nm of step edge, and (q) percentage of clusters on terraces on the oxidized (red) and reduced surfaces (blue). For plots (o–q), we used the average of three STM images at each temperature. The error bars represent the standard deviation between the respective images. (r,s) From an XPS series on separately prepared oxidized (18 ML) and reduced (17 ML) films, we extract (r) the relative Pt 4f XPS peak integrals of the Pt^0^ and Pt^2+^ components, normalized to the total peak integral, and (s) the binding energy of the Pt 4f_7/2_ peak of the Pt^0^ component for the oxidized (red) and reduced (blue) surface. The spectra were measured with Mg Kα radiation in 70° grazing emission (w.r.t. the surface normal). (t) STM image of the CeO_1.94_(111) surface in between the clusters after annealing to 800 K shows O vacancies as partially ordered dark spots. Imaging conditions: *U*_b_ = 3.5 V, *I*_t_ = 1.0 nA.

**Figure 5:**
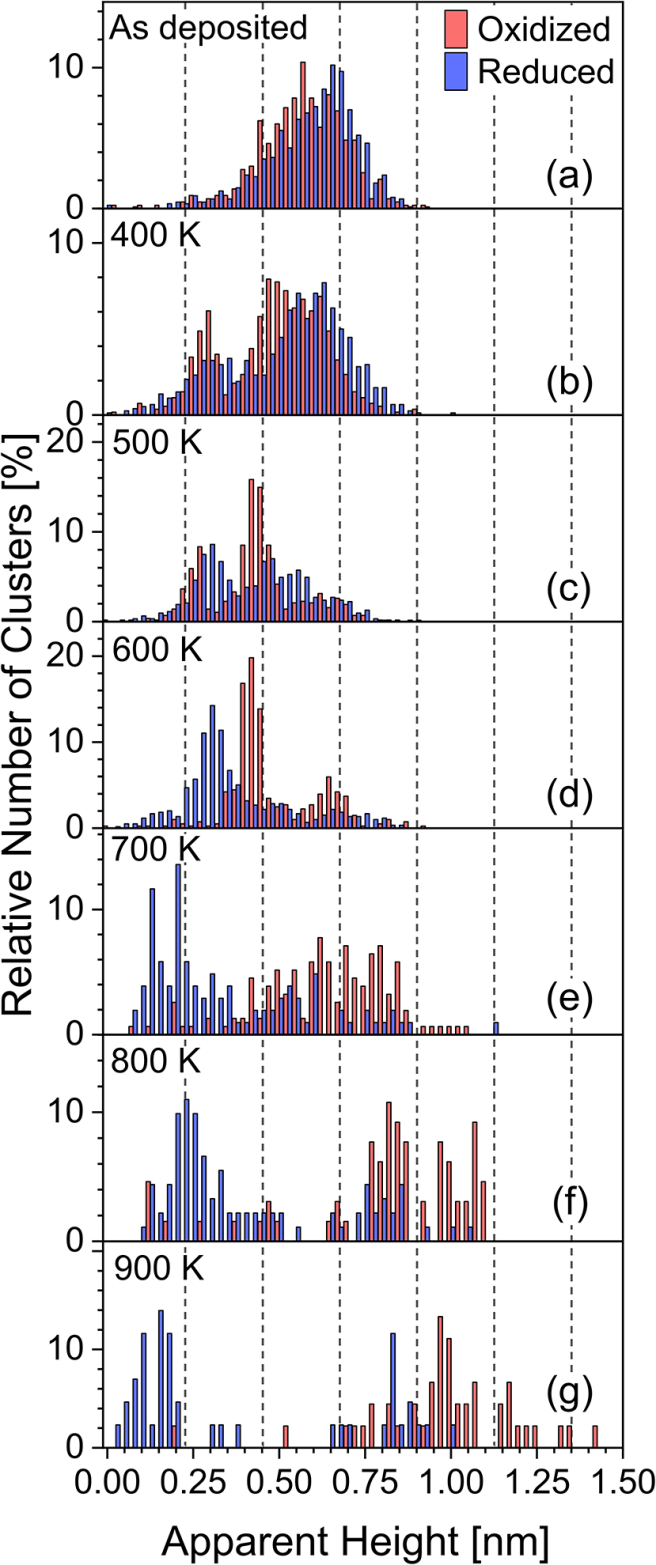
Histograms showing the distribution of apparent cluster heights of as-deposited and sintered Pt_20_ on oxidized (red) and reduced (blue) ceria films, obtained from the STM images in Figure 4, measured (a) directly after cluster deposition and after annealing to (b) 400 K, (c) 500 K, (d) 600 K, (e) 700 K, (f) 800 K, and (g) 900 K. The vertical dashed lines represent the step height of Pt(111) as a guide to the eye.

When the clusters are deposited on the bare oxidized and reduced surfaces, the STM images show a statistical distribution without preferential adsorption at steps (Figure 4a,h). From the apparent cluster height analysis in Figure 5a, it is striking that the histograms, which later will differ drastically, overlap nearly perfectly, with clusters that are between 2 and 3 layers high, irrespective of the number of O vacancies present. Note that for the histograms, the apparent cluster height—which reflects geometrical as well as electronic effects—is referenced to the median of the brightest 20% of the surrounding background to guarantee a constant background level irrespective of the degree of reduction (see Section S1 for more details). Such a 3D cluster structure is in agreement with previously reported calculations for Pt_13_ clusters on stoichiometric ceria.^90^ The XPS measurements on both samples are also the same, once more indicating that the Pt clusters are of a comparable degree of oxidation and unaffected by the presence of the O vacancies. The analysis of the Pt 4f region indicates a Pt^2+^ content of around 10% (see Figure 4r). We therefore conclude that the clusters sit on stoichiometric areas of the support and that the present surface reduction has no long-range effect. The observed Pt oxidation is in line with previous reports from experimental and theoretical studies that Pt particles can donate electrons to ceria, thereby creating Ce^3+^:^17,40^ Lykhach et al. could determine experimentally a charge transfer limit of 1 electron per 10 Pt atoms for particle sizes between 30 and 70 atoms by resonant photoemission.^17^ Similarly, calculations by Bruix et al. found a reduction of up to two Ce^4+^ cations by Pt_8_, again corresponding to a Pt^2+^ content of roughly 10%.^91^

If we anneal the clusters to 400 K, the first change in morphology arises (Figures 4b,i and 5b). On both supports, the cluster height distributions show a new, lower species, at just above 1 layer in height, containing approximately 1 of the clusters, independent of the O vacancy coverage in ceria. We note that this change occurs while the clusters do not exhibit significant mobility: No cluster migration to steps is observed, and their cluster coverage remains unchanged within the error bars (Figure 4o). Such a shift in apparent height of a significant number of the clusters is thus most likely related to a flattening upon lattice oxygen reverse spillover or CO desorption. There are good arguments for both: The Pt 4f XPS spectra show a continuous decrease in binding energy for the overall Pt 4f signal, as shown in Figure 4s, which is similar to the signature for CO desorption we have observed in TPD measurements on Pt_19_/Fe_3_O_4_(001).^92^ In addition, lattice oxygen reverse spillover has been reported at this temperature for Pt nanoparticles on ceria^32,93^ and in the case of Pt_6_ clusters on ceria, Negreiros et al. have found a flattening upon the spillover process to be slightly exothermic in a DFT study.^40^ That being said, STM does not distinguish between true morphological changes and differences in the density of states.

This flattening of the clusters continues after the 500 K annealing step (Figures 4c,j and 5c), but now, the two samples begin to differ. On the oxidized support, we see a distinct isomer transition where a distribution of higher clusters transforms into a sharp two-layer cluster peak. On the reduced surface, in contrast, we see a peak just above one layer in height beginning to grow at the expense of larger clusters.

After annealing to 600 K, the differences between the two samples become more distinct, as shown in Figures 4d,k and 5d. First, on both surfaces, the isomer that was already dominant at 500 K grows further. At the same time, both height distributions start to broaden, from a sub-single layer up to four layers in height. This broadening is a classical signature for Ostwald ripening, where larger clusters grow at the expense of smaller ones by size-dependent atom detachment rates.^94^ At the same time, the expected concomitant decrease in cluster coverage is still small since few clusters are fully dissolved yet. The position of the Pt 4f peak shifts by −0.4 eV for the oxidized and −0.3 eV for the reduced sample (Figure 4s) as a result of the changed electron hole screening for the sintered clusters, a well-known final state effect.^18,95^ The fact that this ripening mechanism sets in around the Hüttig temperature (608 K for Pt), where atoms start to detach from undercoordinated sites,^96^ suggests that the atom detachment is significantly activated, e.g., not facilitated by strong atom–support interactions. At this onset of atom diffusion, we observe a second distinct difference associated with the support oxidation state, namely, a clear increase in the relative Pt^2+^ peak in the XPS Pt 4f region of the oxidized sample only (see Figure 4o), beyond the constant Pt^2+^ level associated with the charge transfer at the cluster-support interface, which we discussed above. We interpret this increase—in line with the redispersion process described in the literature^23,24,97,98^ —as indicative of the diffusion of some of these Pt atoms to steps, where excess lattice oxygen helps to incorporate them as Pt^2+^ species. This Pt^2+^ increase from ∼10 to ∼20% is only intermediate and returns to the previous level in the next temperature step.

At higher temperatures (Figure 4e–g,l–n and 5e–g), the cluster height distributions start to diverge even more dramatically, resulting in a completely different cluster dimensionality on the two supports: On the oxidized sample, the Ostwald ripening results in a classical increase in the cluster heights, up to 3–4 layers high at 700 K and to 5–6 layers high at 900 K. On the reduced surface, in contrast, the distribution is now dominated by clusters of only one layer in height or lower. We thus have a nearly exclusively 3D cluster growth on the oxidized and a predominantly 2D cluster formation on the reduced support. Since the cluster coverage on both surfaces is similar (Figure 4o), they must, on average, contain a similar number of Pt atoms. The 2D clusters on the reduced support thus have a diameter significantly larger than the 3D ones on the oxidized support. Note that throughout the annealing experiments, there is no indication for cluster diffusion and related Smoluchowski ripening, as the number of clusters trapped at step edges is constant (Figure 4p). The relative number of clusters on terraces (with respect to the total number of clusters) start to decrease from 600 K (Figure 4q). In combination, these coverage trends suggest that the sintering process almost exclusively takes place on terraces up to 800 K. As a result, increasing the number of step edges might lead to effective cluster stabilization, thus mostly preventing sintering.

Our results raise the question as to why such a relatively small difference in the O vacancy concentration has such a dramatic influence on the cluster geometry. One possible explanation might be the nucleation of entirely new 2D clusters on the O vacancies. From a theoretical study by Salichon et al., it is known that Pt atoms can be stabilized over O vacancies.^19^ Furthermore, in an experimental study by Zhou et al., the cluster coverage upon thermal deposition of Pt atoms scales with the number of O vacancies, which act as nucleation centers.^18^ In contrast, Negreiros and Fabris report that O vacancies do not act as trapping centers for Pt_4_ or Pt_6_ clusters.^40^ In our experiments, it is striking that although a slight oxidation state difference in both of the two ceria films still persists at higher temperatures (Figure S10), the cluster coverage trend is identical (within the error bars) on both supports from 700 K onward, when 80% of the clusters have already been lost (Figure 4o). It is thus highly improbable that the dimensionality effect stems from a complete redispersion of our Pt_20_ into atoms and the subsequent nucleation of completely new clusters. Our observations further imply that the atom detachment energy and probability are independent of support reduction and do not scale with the interface area between cluster and surface—even though the overall cluster morphology seems to be highly dependent on the cluster-support interaction strength. We conclude that the initially deposited clusters must have flattened during the annealing at intermediate temperatures, but exclusively on the reduced support.

Rather than leading to nucleation of new clusters, the O vacancies hence seem to induce the flat binding geometry of the clusters by either their presence under the clusters or at the rim, strengthening the cluster-support interaction. Most likely, the O vacancies have migrated to these positions, since the low coverage of both, Pt clusters and O vacancies, would not statistically lead to a significant interaction. It has been suggested that ceria lattice strain can strongly influence the aggregation or repulsion of O vacancies,^36,99^ which could explain the stabilization of multiple O vacancies at the slightly reduced ceria under clusters in our results. This reordering of the O vacancies is supported by an observed change in STM background on the reduced sample as well as more stable scanning conditions from the 700 K annealing step on. While the background appeared widely corrugated, streaky, and highly interactive with the STM tip at the previous temperatures (Figure 4h–k)—indicating a high O and thus vacancy mobility—it is now flatter and instead exhibits a fine structure which persists up to 900 K (Figure 4l–n). This fine structure appears to be comparable to that we have observed in STM images after the UHV reduction step during typical film preparation (Figure 2b), which indicates an ordered O vacancy structure. Indeed, an atomic-scale close-up of the surface after annealing to 800 K, shown in Figure 4t reveals that the presence of O vacancies (dark spots) is still observed in areas between the clusters. Strikingly, from 700 K on, some of the clusters are less than one layer high, which might point to an embedded structure, as reported previously for Pt particles on ceria powders.^100,101^ In our STM images, we observe that some of these flat clusters exhibit a slightly hexagonal shape (Figure S11), whereby two different orientations occur that are rotated by 30° with respect to each other. These orientations could imply that different ceria edges are interfacing embedded Pt clusters, such as the two sets of step edges reported by Nilius et al. for ceria islands.^83^

Two-dimensional Pt nanoparticles have been observed previously on ceria or ceria/alumina powder catalysts. Their preferential formation was either explained by the nature of the support facet^101^ or the presence of O vacancies due to reductive treatment.^100^ More generally, also on an irreducible MgO support, where O vacancies lead to color centers, it has been reported that the introduction of such vacancy defects provides charges that stabilize flatter cluster geometries, in this case for Au_20_ clusters.^102^ In both the Au_20_ on MgO and the Pt nanoparticles on CeO_2_ cases, a more facile CO oxidation is found concomitantly with the change to more 2D structures. Overall, our combined STM and XPS investigation provides a detailed atomic-scale picture of Pt cluster sintering from around 600 K, where O vacancies stabilize highly wetted, 2D clusters and provide a platform for future investigations regarding potential differences in catalytic activity.

## 4 Conclusion

In conclusion, we have presented a new approach to tuning the dimensionality of Pt clusters, namely, by controlling the O vacancy coverage of a ceria support. For this purpose, we have prepared atomically flat, closed CeO_2_(111) thin films on a Rh(111) substrate with an oxidized, stoichiometric, and reduced surface, respectively. X-ray diffraction measurements show that the ceria (111) films grow single crystalline with a single domain. C^18^O TPD measurements confirm that our films are essentially fully closed, which enables future measurements of the catalytic activity of clusters on these supports.

Size-selected Pt_20_ clusters initially exhibit the same shape and oxidation state on the oxidized and slightly reduced ceria supports directly after deposition. Here, using size-selected clusters presents a key advantage over cluster formation from deposited atoms, as the deposited particles are statistically distributed rather than nucleated at defects, thus allowing us to study the influence of O vacancies on the cluster sintering process independently. The first effect is observed around 400 to 500 K, where the clusters appear lower in STM due to lattice oxygen reverse spillover changing their preferred geometry; cluster distributions appear the same on both supports. Subsequently, Ostwald ripening sets in around 600 K on both samples, leading to the detachment of single Pt atoms and a broadening of the cluster size distribution. Some of these Pt atoms can intermittently be captured as Pt^2+^ species on step edges on the stoichiometric ceria support, where sufficient numbers of O atoms are available for their stabilization. While the extent of sintering is surprisingly similar on both supports, indicating that the clusters are not completely dissolved and reformed on nucleation sites, the cluster shape starts to differ drastically: 2D-island-like on the reduced and 3D-spherical on the oxidized support. Our results suggest that the O vacancies can diffuse to the clusters during the sintering onset and thus stabilize a wetted geometry by modifying the Pt-ceria interface energy. We further conclude from our experiments that the atom exchange only occurs between clusters on the same terraces, suggesting that a high step density may be used to stabilize clusters against sintering.

As discussed, several previous reports show a link between cluster dimensionality and catalytic activity. In this fundamental work on a highly defined Pt/ceria model catalyst, we have explored a set of parameters that can steer cluster dimensionality and thus facilitate activity tuning in particle-mediated catalysis, in particular step density, oxidation state, and defect distribution of the support. In an optimal parameter window that mitigates cluster sintering while allowing oxidation state modulation by reactive gas atmospheres, even cluster fluxionality might become accessible experimentally.

## Supplementary Material



## Notes

Background definition for apparent cluster height quantification; quantification of the Ce^3+^/Ce^4+^ content via XPS; apparent height profiles of CeO_1.58_ islands on Rh(111) from an STM image; STM image of the O(2 × 1) adlayer on Rh(111) next to CeO_1.58_ islands; STM image of ordered O vacancies on a closed CeO_1.7_ film; energy-dependent LEED patterns of a surface-reduced CeO_1.94_ film; STM images of a closed, stoichiometric CeO_2_ film; STM images of a CeO_2–x_ film before and after CH_3_OH reduction; fitted Pt 4f XPS spectra after each annealing step of a temperature series; Ce 3d XPS spectra after each annealing step of a temperature series; and STM images showing the hexagonal structure of sintered, flat Pt clusters after annealing to 800 and 900 K (S11) (PDF)
