# Supplementary material for: Steering Pt Cluster Dimensionality via the Surface Oxidation State of CeO2(111) Thin Films
Source: ACS Catal. 2025 Oct 22;15(21):18369–82. doi: 10.1021/acscatal.5c05570 (PMC12603995; doi:10.1021/acscatal.5c05570)
Supplement: Supplementary file 1 [file cs5c05570_si_001.pdf]

# Supporting Information

for

# Steering Pt Cluster Dimensionality via the Surface Oxidation State of $CeO_2(111)$ Thin Films

Johanna Reich,[1] Mina Soltanmohammadi,[2] Vedran Vonk,[3] Sebastian Kaiser,[1,2] Ueli Heiz,[2] Andreas Stierle,[3,5] Friedrich Esch*,[2] and Barbara A.J. Lechner*,[1,4]

[1] Functional Nanomaterials Group and Catalysis Research Center, Department of Chemistry, TUM School of Natural Sciences, Technical University of Munich, 85748 Garching, Germany.

[2] Chair of Physical Chemistry and Catalysis Research Center, Department of Chemistry, TUM School of Natural Sciences, Technical University of Munich, 85748 Garching, Germany.

[3] Centre for X-Ray and Nano Science, Deutsches Elektronen-Synchrotron (DESY), D-22607 Hamburg

[4] Institute for Advanced Study, Technical University of Munich, 85748 Garching, Germany.

[5]Physics Department, University of Hamburg, D-20355 Hamburg, Germany

*Corresponding authors: friedrich.esch@tum.de, bajlechner@tum.de

## S1 Background definition for apparent cluster height quantification

To determine the apparent cluster heights from STM images, it is crucial to select a correct background reference level. The cluster detection occurs with an automatic detection algorithm, which detects all features above a certain height threshold. While, for the cluster, we simply use the maximum intensity of the detected feature (after applying a 3x3 Gauss filter to mitigate spike values), there are many options to select the background level in a way that it is independent of the degree of reduction of the surface (imaged as depressions) and not affected by other defects such as screw dislocations that hamper simple terrace referencing. In Figure S1, we compare different background reference methods that all apply median values, since the arithmetic mean is more strongly influenced by imaging artifacts like streaks or small tip crashes. However, even the median might be influenced by the nature of the scanned surface: If the surface is corrugated, like it is the case at the lower annealing temperatures in Figure 4 in the main article, using the median of the overall terrace background ("outside all particles") is not applicable to all clusters. This is visible in Figure S1 (a), where the apparent height distribution is significantly broadened and shifted towards each other between the oxidized and reduced support. Therefore, we identify an improved background reference around each cluster individually ("around each particle"), as shown in Figure S1 (b). In this case, the shift slightly decreases, but is still influenced by the high background corrugation of the reduced surface.

The truest background level is that of the stoichiometric, pristine ceria, while vacancies introduce apparent deviations from this reference, e.g. in the form of dark vacancies. Since the reduced ceria surface in this work contains a significant amount of dark areas in many images, we apply the median only to the 20% highest pixels around the cluster. Indeed, in Figure S1 (c)) we see a rather perfect match for the height distribution of the Pt clusters on the two supports. To check the validity of this strategy, we compare with the lowest 20% of pixels around each particle (Figure S1 (d)) and again find the same offset for the reduced surface as in (a) and (b). We hence use the median of the highest 20% of pixels around each particle for the height distribution evaluation in the annealing series in Figure 5.

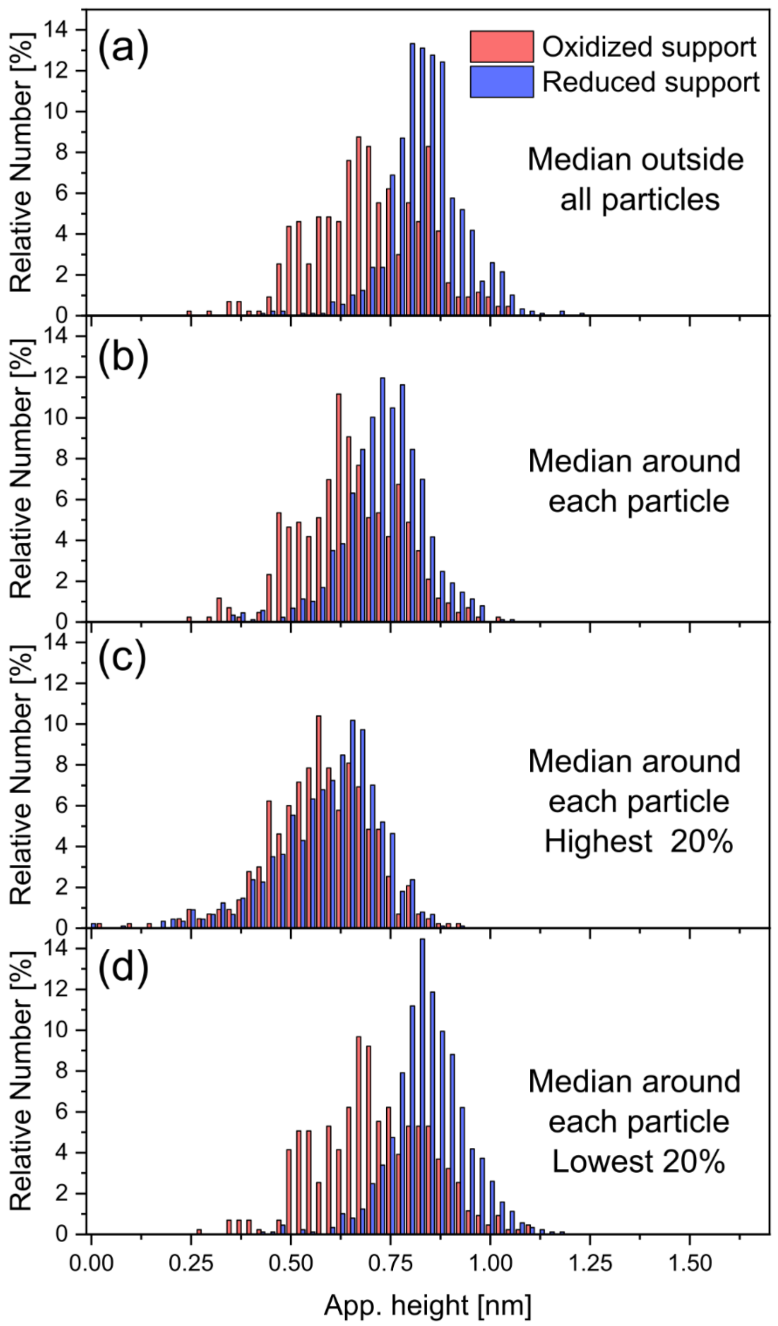


**Figure S1** Apparent height distribution of as deposited $Pt_{20}$ on oxidized $CeO_2$ and reduced $CeO_{1.94}$ thin films on Rh(111) using different background correction methods on the identical STM data: (a) The median outside all particles, (b) the median around each particle, (c) the median of the highest and (d) lowest 20% of pixels around each particle.

## S2 Quantification of the $Ce^{3+}$/$Ce^{4+}$ content from normal and grazing emission XPS

The quantification of the $Ce^{3+/4+}$ content in ceria thin films from Ce 3d XPS spectra is challenging. The spectrum consists of three doublets corresponding to $Ce^{4+}$, which are typically named u+v, u"+v" and u'"+v'", whereby the u peaks correspond to Ce $3d_{3/2}$ and the v peaks to Ce $3d_{5/2}$. Two further doublets exist for $Ce^{3+}$, which are denoted as $u_0$+$v_0$ and u'+v' [1–3]. To quantify the respective contributions of the different oxidation states, there are several methods available: (i) classical fitting of all peaks and integration of $Ce^{3+}$ and $Ce^{4+}$ contributions, (ii) linear combination of fully oxidized and fully reduced spectra to fit spectra of an unknown oxidation state, and (iii) integration of the specific Ce $3d_{3/2}$ u'" peak, which is well separated from the other peaks, around 916.8 eV, and arises solely from $Ce^{4+}$. Classical peak fitting works sufficiently well for spectra measured in normal emission on several ML thick films. We apply the fitting parameters from ref. [3] with a tolerance of ± 0.2 eV in the peak position while keeping the peak splitting fixed. A typical fit of a stoichiometric $CeO_2$(111) film is shown in Figure S2. Since we measure with a non-monochromatized X-ray source, we add satellite peaks to account for the Al $K\alpha_3$ and Al $K\alpha_4$ radiation at -9.8 and -11.8 eV with respect to the original peaks, respectively [4]. When it comes to thinner films and measuring in grazing emission, both conditions have an influence on the shape of the background caused by altered inelastic scattering of electrons [5]. As a consequence, we cannot reasonably fit those spectra with the fitting parameters mentioned above. The most accurate alternative would be the linear combination method, which includes measuring one fully oxidized $CeO_2$ and one fully reduced $Ce_2O_3$ spectrum under the same conditions and fitting a spectrum of unknown oxidation state as a sum of both components, where the fitted ratio gives the degree of reduction. However, this method requires fully oxidized and reduced reference spectra for each measurement condition. Unfortunately, it has been impossible to produce pure $Ce_2O_3$ reference films, especially in the case of thicker films. Thus, we decided to determine the oxidation state exclusively via the $Ce^{4+}$contribution of the Ce $3d_{3/2}$ u'" component.

For this procedure, we measure a suitable, maximally oxidized reference sample in normal and grazing emission with the same measurement parameters as the unknown samples. We determine the residual $Ce^{3+}$ trace in the oxidized reference spectrum (typically <1%) by fitting the complete normal emission spectrum with all components. We assume that it is distributed homogeneously and thus present in the same amount in the corresponding grazing emission spectrum which we cannot fit adequately. We then fit only the Ce $3d_{3/2}$ u'" component with a

simple Voigt peak shape, normalize its integral to the integral of the entire Ce 3d spectrum (Shirley background-corrected) and establish with these normalized values the reference for a fully (>99%) oxidized sample. The unknown samples are treated analogously and the normalized Ce $3d_{3/2}$ u''' peak integral is used to calculate the $Ce^{4+}$ loss due to reduction. the unknown spectrum is then normalized to the same integral as the reference.

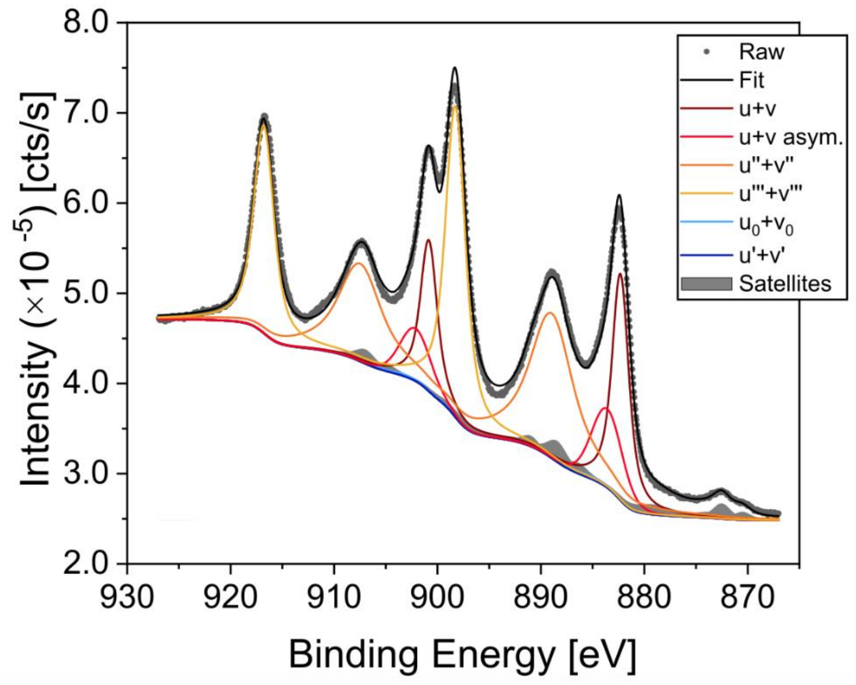


**Figure S2** Ce 3d XPS spectrum of a fully oxidized (0.7% $Ce^{3+}$), 14 ML thick $CeO_2(111)$ film on Rh(111), fitted with the parameters from Skála et al. [3], measured with Al Kα radiation in normal emission. The black shaded areas represent Al $K_{\alpha 3}$ and $K_{\alpha 4}$ X-ray satellites arising from our non-monochromatized X-ray source.

## S3 Apparent height profiles of $CeO_{1.58}$ islands on Rh(111)

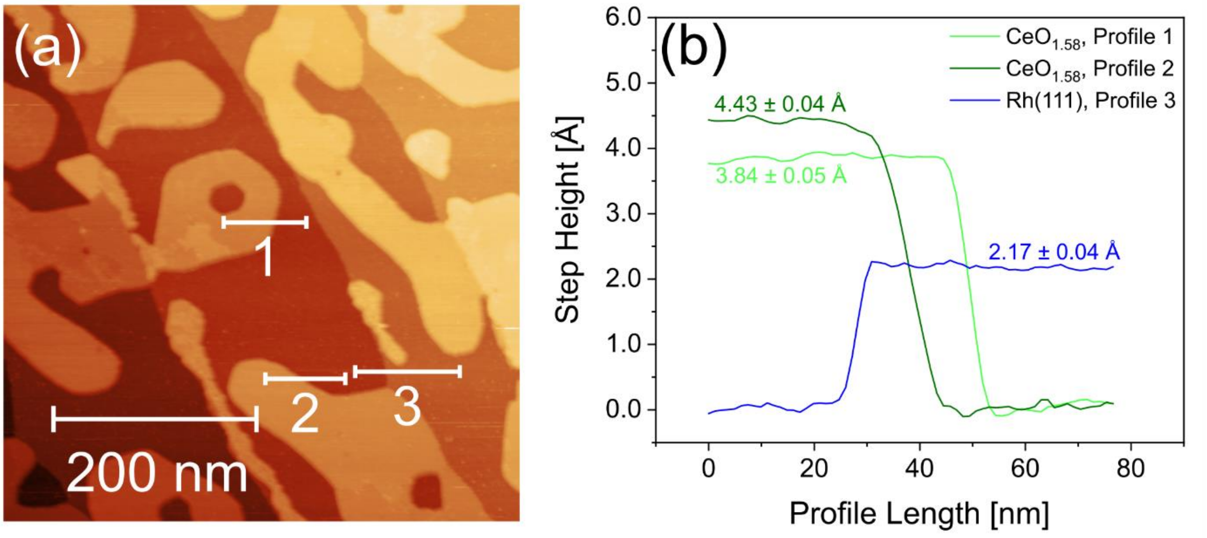


**Figure S3** (a) STM image of $CeO_{1.58}$ islands (brighter areas) on Rh(111) (darker areas). *Imaging conditions*: $U_b$ = 1.5 V, $I_t$ = 300 pA. (b) The apparent height along the line profiles 1, 2 and 3 is shown, where profiles 1 and 2 represent the height of $CeO_{1.58}$ islands. The given standard deviation corresponds to the spreading of the measured values on top and below the island. Profile 3 was taken across a Rh(111) step and fits well with the Rh(111) step height from literature [6].

## S4 O(2×1) adlayer on Rh(111) next to $CeO_{1.58}$ islands

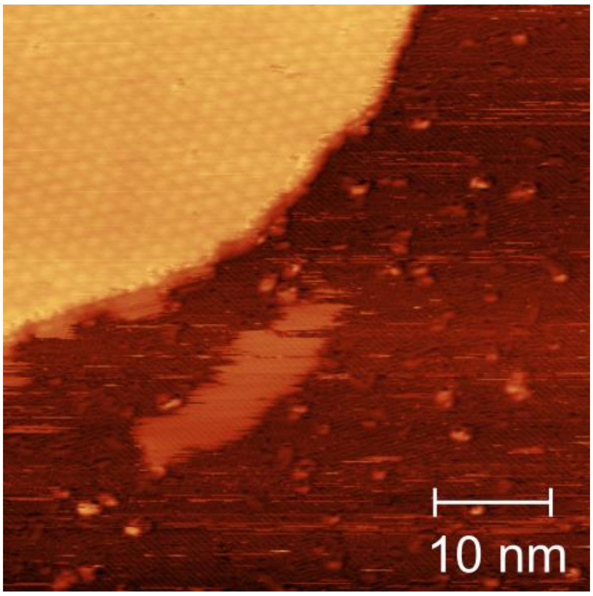


**Figure S4** STM image of $CeO_{2-x}$(111) islands on Rh(111). *Imaging conditions*: $U_b$ = 1.5 V, $I_t$ = 500 pA. The bright area with the Moiré structure on the upper left of the image represents a ceria island, while the darker area in the rest of the image represents the Rh(111) surface. The ordered, partially rotated lines on the Rh surface indicate an O(2×1) adlayer [7,8], present on the surface in different orientations, in agreement with the corresponding LEED pattern.

## S5 Ordered O vacancies on thick $CeO_{1.7}(111)$

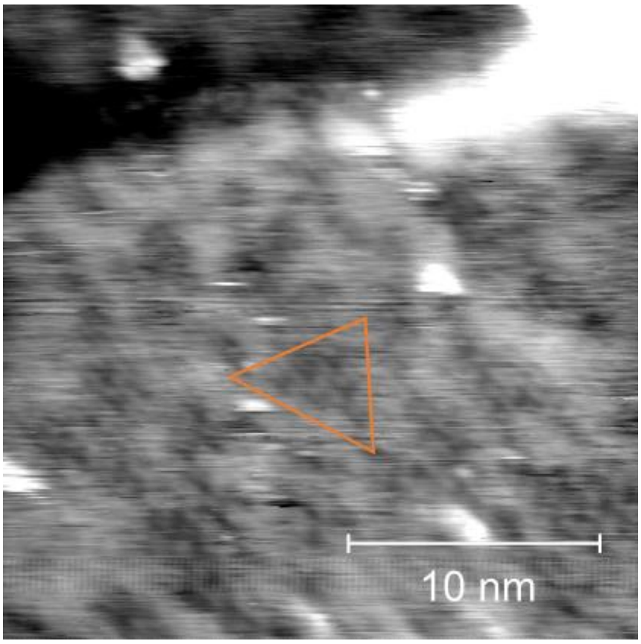


**Figure S5** Room temperature STM image of the 13 ML thick $CeO_{1.7}(111)$ film on Rh(111) after annealing to 1073 K in UHV for 45 min. Between large areas of streaky and cloudy features, we observe areas of ordered, threefold depressions, which resemble oxygen vacancies, most likely arranged in coexisting domains of a $(\sqrt{7}\times\sqrt{7})R19.1°$ and a (3×3) structure [9,10] *Imaging conditions*: $U_b = 3.5$ V, $I_t = 5.0$ nA.

## S6 Energy-dependent LEED patterns of a surface-reduced $CeO_{1.94}$ film

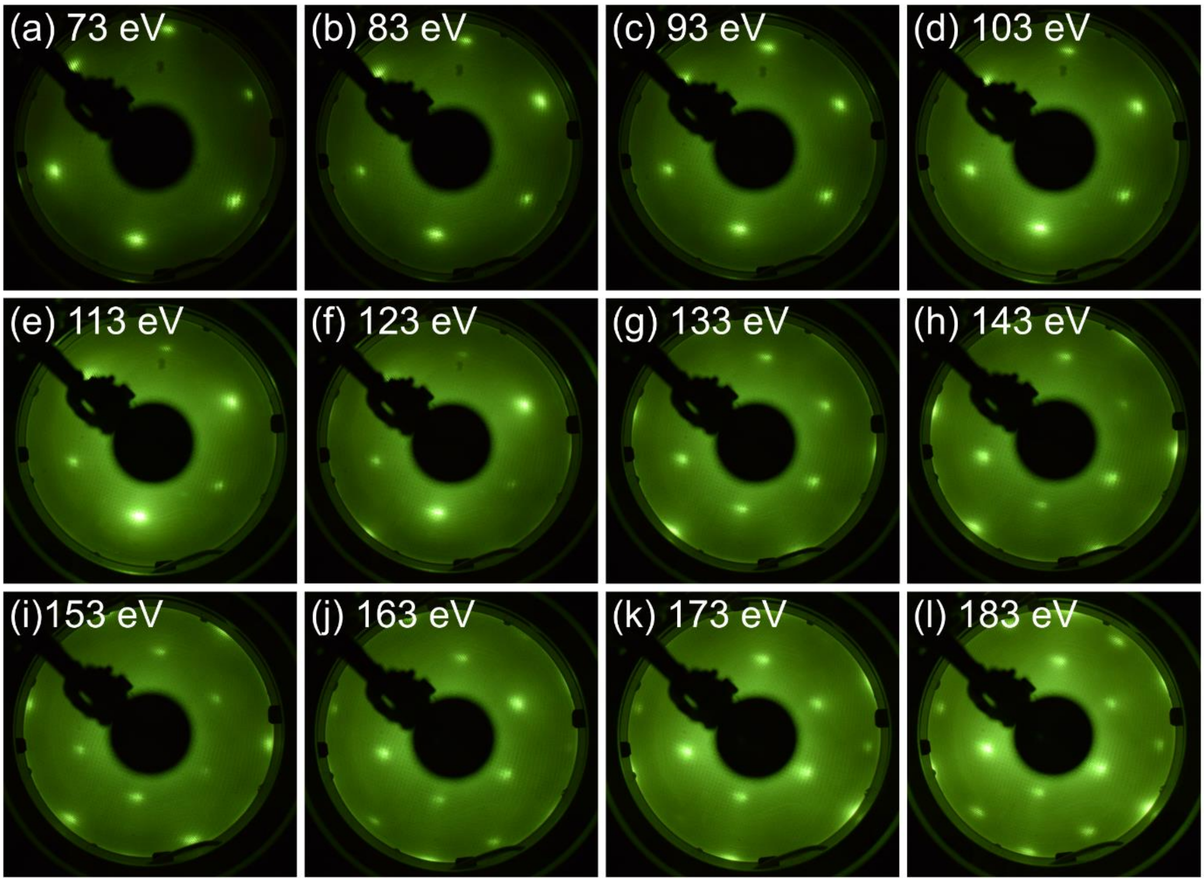


**Figure S6** Energy-dependent LEED patterns of a 20 ML thick, slightly reduced $CeO_{1.94}(111)$ film on Rh(111). This surface was reduced with $CH_3OH$, which acts exclusively on the surface. We can thus assume that the bulk structure is stoichiometric $CeO_2$. While certain energies (e.g. 93 eV, 133 eV) seem to suggest hexagonal symmetry, other energies (in particular 123 eV) clearly show a threefold symmetry. The LEED data thus confirms crystalline $CeO_2$ of a single orientation. As we sweep the energy and thus probe different layers, the two subsets of three bright spots are selected alternately.

## S7 Larger-scale structure of thick, stoichiometric $CeO_2(111)$ films

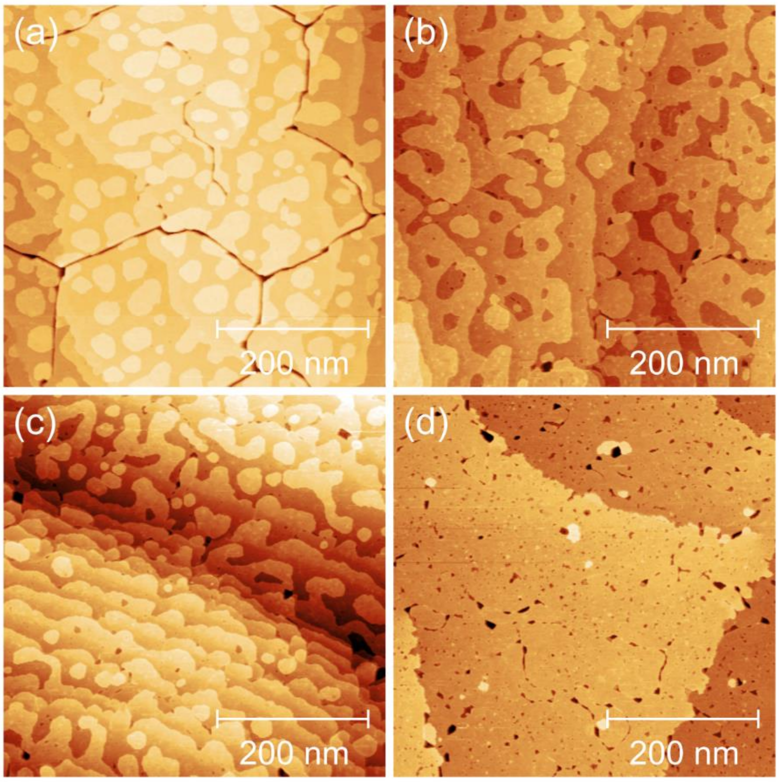


**Figure S7** STM images of an oxidized 13 ML thick $CeO_2(111)$ film on macroscopically different spots of the same sample, roughly 3.5 mm apart from each other. *Imaging conditions*: $U_b$ = 3.5 V, $I_t$ = 300 pA. The long-range structure of the film seems to depend strongly on the structure of the underlying Rh(111) support. Interestingly, even though the film is 13 ML thick and the influence from the substrate should be minimal, it still appears somewhat strained, evident in the approximately hexagonally arranged deep cracks between large terraces overgrown by small islands (a), and randomly distributed holes (d). This might be due to the relatively fast (> 2 K/s) cooling rate after the annealing steps. It seems that the formation of cracks and holes decreases with an increasing number of steps (b,c).

## S8 Larger-scale structure of a $CeO_{2-x}$ film before and after reduction with $CH_3OH$

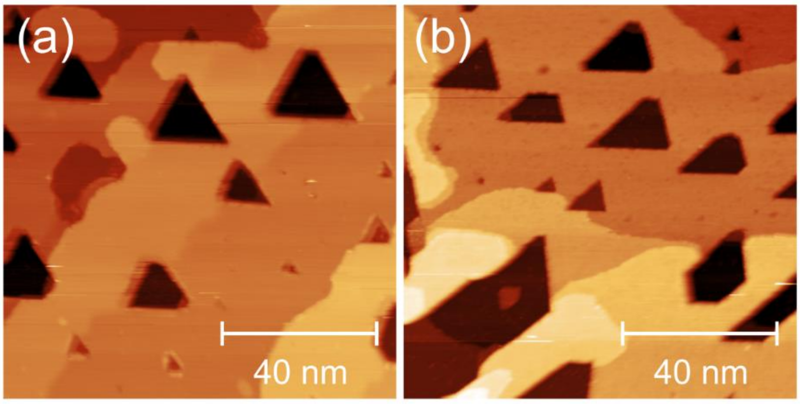


**Figure S8** STM images of a 4.7 ML thick $CeO_{2-x}(111)$ film (a) as prepared and (b) after two reduction cycles, each consisting of dosing 10 L $CH_3OH$ at room temperature, followed by a 1 K/s heating ramp in UHV to 600 K and keeping this temperature for 5 min. In these large-scale images, the surface does not show any clear difference in morphology. *Imaging conditions*: (a) $U_b$ = 3.5 V, $I_t$ = 300 pA and (b) $U_b$ = 2.0 V, $I_t$ = 300 pA.

## S9 Fitted Pt 4f spectra during a stepwise annealing series

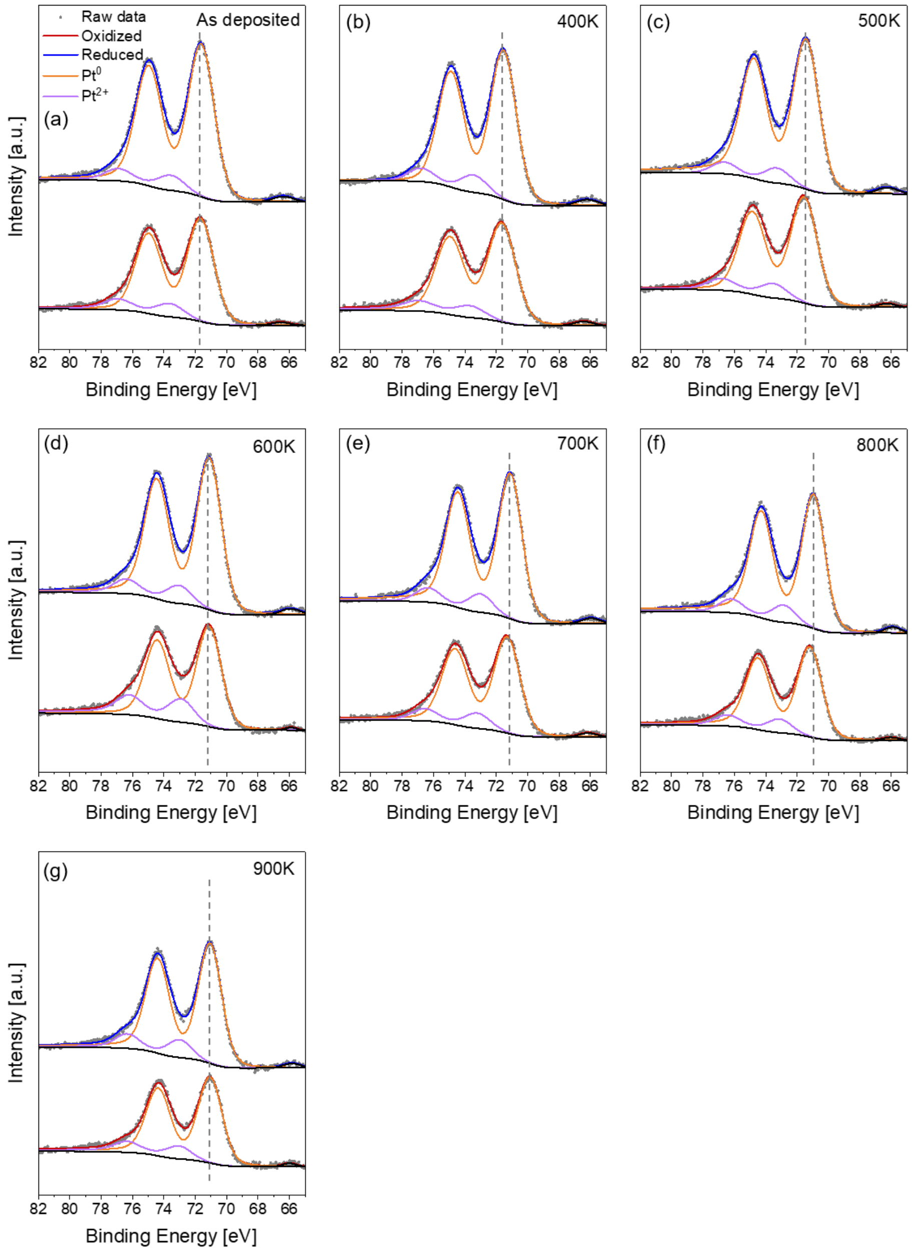


**Figure S9** Pt 4f XPS spectra of the 18 ML thick, oxidized (red) and 17 ML thick, reduced (blue) $CeO_{1.94}(111)$ thin films and their fits throughout the whole temperature series applied for investigating the $Pt_{20}$ sintering behavior and morphology changes in Figure 4. The spectra are measured in 70° grazing emission with MgKα X-ray radiation. The grey dashed lines represent the position of the Pt $4f_{7/2}$ component of the reduced spectrum as a guide to the eye.

## S10 Ce 3d XPS spectra during a stepwise annealing series

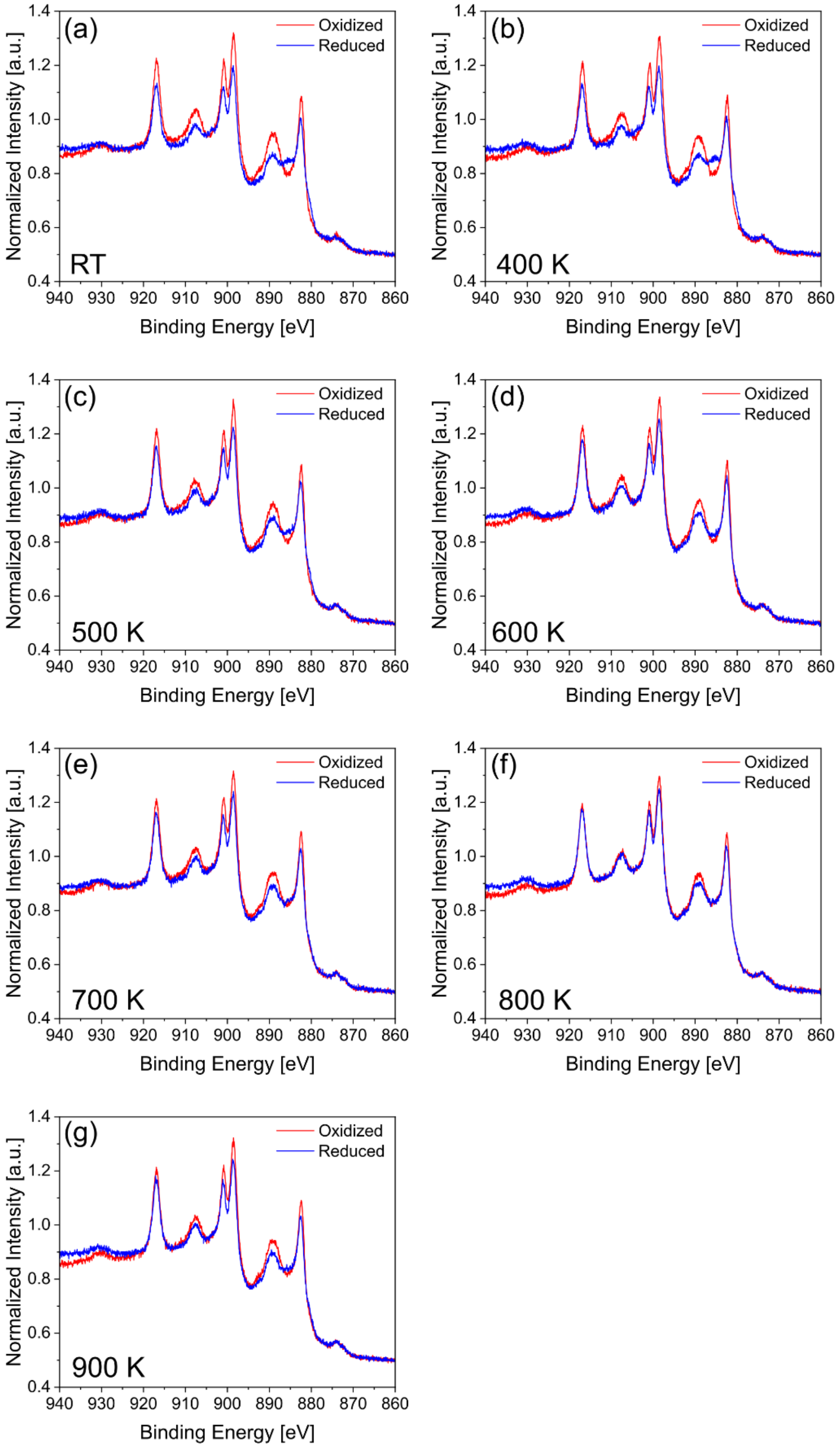


**Figure S10** Ce 3d XPS spectra of the 18 ML thick, oxidized (red) and 17 ML thick, reduced (blue) $CeO_{1.94}(111)$ thin films throughout the whole temperature series applied for investigating the $Pt_{20}$ sintering behavior and morphology changes in Figure 4. The spectra are measured in

70° grazing emission with MgKα X-ray radiation. The most significant difference in the spectra is visible around 885-886 eV, where the $Ce3d_{5/2}$ component of the u'+v' component ($Ce^{3+}$) is located. Even though the difference is slowly equaling out over the whole temperature range, a slight difference persists up to 900 K.

## S11 Hexagonal structure of sintered, flat Pt clusters

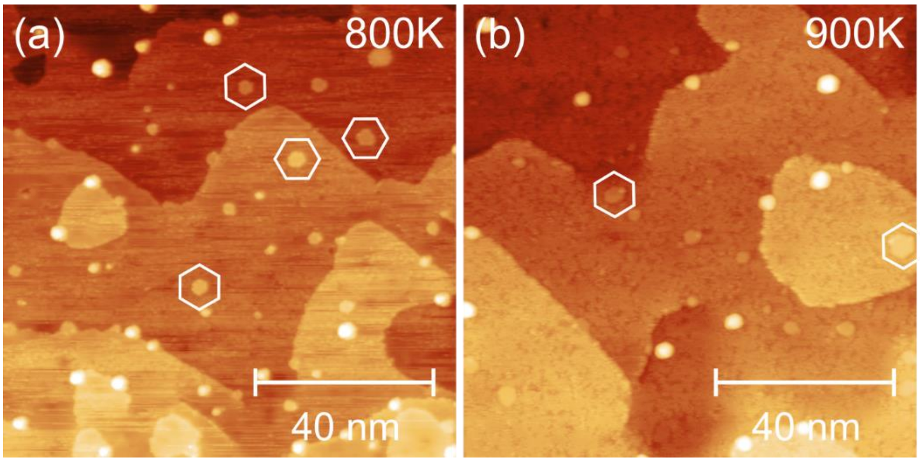


**Figure S11** Examples for hexagonally shaped clusters in STM images, measured after annealing the $Pt_{20}$ clusters on the reduced $CeO_{2-x}(111)$ film surface to (a) 800 K. and (b) 900 K. They appear in two different configurations, which are rotated towards each other by 30°. *Imaging conditions*: (a) $U_b = 3.0$ V, $I_t = 200$ pA and (b) $U_b = 3.5$ V, $I_t = 200$ pA

## References


(1) Burroughs, P.; Hamnett, A.; Orchard, A. F.; Thornton, G. Satellite Structure in the X-Ray Photoelectron Spectra of Some Binary and Mixed Oxides of Lanthanum and Cerium. *J.C.S., Dalt. Trans.* **1976**, No. 1686–1698.

(2) Pfau, A.; Schierbaum, K. D. The Electronic Structure of Stoichiometric and Reduced $CeO_2$ Surfaces: An XPS, UPS and HREELS Study. *Surf. Sci.* **1994**, *321*, 71–80.

(3) Skála, T.; Šutara, F.; Škoda, M.; Prince, K. C.; Matolín, V. Palladium Interaction with $CeO_2$, Sn-Ce-O and Ga-Ce-O Layers. *J. Phys. Condens. Matter* **2009**, *21*, 055005.

(4) Moulder, J. F.; Stickle, W.; Sobol, P. E.; Bomben, K. D. *Handbook of X-Ray Photoelectron Spectroscopy*; Physical Electronics: Minnesota, 1992.

(5) Tougaard, S. Practical Guide to the Use of Backgrounds in Quantitative XPS. *J. Vac. Sci. Technol. A* **2021**, *39*, 011201.

(6) Stempel, S.; Bäumer, M.; Freund, H. J. STM Studies of Rhodium Deposits on an Ordered Alumina Film - Resolution and Tip Effects. *Surf. Sci.* **1998**, *402–404*, 424–427.

(7) Gustafson, J.; Mikkelsen, A.; Borg, M.; Lundgren, E.; Köhler, L.; Kresse, G.; Schmid, M.; Varga, P.; Yuhara, J.; Torrelles, X.; Quirós, C.; Andersen, J. N. Self-Limited Growth of a Thin Oxide Layer on Rh(111). *Phys. Rev. Lett.* **2004**, *92* (12), 126102.

(8) Farber, R. G.; Turano, M. E.; Oskorep, E. C. N.; Wands, N. T.; Iski, E. V.; Killelea, D. R. The Quest for Stability: Structural Dependence of Rh(111) on Oxygen Coverage at Elevated Temperature. *J. Phys. Chem. C* **2017**, *121*, 10470–10475.

(9) Wilkens, H.; Schuckmann, O.; Oelke, R.; Gevers, S.; Schaefer, A.; Bäumer, M.; Zoellner, M. H.; Schroeder, T.; Wollschläger, J. Stabilization of the Ceria $\iota$-Phase ($Ce_7O_{12}$) Surface on Si(111). *Appl. Phys. Lett.* **2013**, *102*, 111602.

(10) Duchoň, T.; Dvořák, F.; Aulická, M.; Stetsovych, V.; Vorokhta, M.; Mazur, D.; Veltruská, K.; Skála, T.; Mysliveček, J.; Matolínová, I.; Matolín, V. Ordered Phases of Reduced Ceria as Epitaxial Films on Cu(111). *J. Phys. Chem. C* **2014**, *118*, 357–365.
